# dsMTL: a computational framework for privacy-preserving, distributed multi-task machine learning

**DOI:** 10.1093/bioinformatics/btac616

**Published:** 2022-09-08

**Authors:** Han Cao, Youcheng Zhang, Jan Baumbach, Paul R Burton, Dominic Dwyer, Nikolaos Koutsouleris, Julian Matschinske, Yannick Marcon, Sivanesan Rajan, Thilo Rieg, Patricia Ryser-Welch, Julian Späth, The COMMITMENT Consortium, Carl Herrmann, Emanuel Schwarz

**Affiliations:** Department of Psychiatry and Psychotherapy, Central Institute of Mental Health, Medical Faculty Mannheim, Heidelberg University, Mannheim 68158, Germany; Health Data Science Unit, Medical Faculty Heidelberg & BioQuant, Heidelberg 69120, Germany; Chair of Computational Systems Biology, University of Hamburg, Hamburg 22607, Germany; Computational Biomedicine Lab, Department of Mathematics and Computer Science, University of Southern Denmark, Odense 5230, Denmark; Population Health Sciences Institute, Newcastle University, Newcastle upon Tyne NE2 4AX, UK; Department of Psychiatry and Psychotherapy, Section for Neurodiagnostic Applications, Ludwig-Maximilian University, Munich 80638, Germany; Department of Psychiatry and Psychotherapy, Section for Neurodiagnostic Applications, Ludwig-Maximilian University, Munich 80638, Germany; Chair of Computational Systems Biology, University of Hamburg, Hamburg 22607, Germany; Epigeny, St Ouen, France; Department of Psychiatry and Psychotherapy, Central Institute of Mental Health, Medical Faculty Mannheim, Heidelberg University, Mannheim 68158, Germany; Department of Psychiatry and Psychotherapy, Central Institute of Mental Health, Medical Faculty Mannheim, Heidelberg University, Mannheim 68158, Germany; Population Health Sciences Institute, Newcastle University, Newcastle upon Tyne NE2 4AX, UK; Chair of Computational Systems Biology, University of Hamburg, Hamburg 22607, Germany; Health Data Science Unit, Medical Faculty Heidelberg & BioQuant, Heidelberg 69120, Germany; Department of Psychiatry and Psychotherapy, Central Institute of Mental Health, Medical Faculty Mannheim, Heidelberg University, Mannheim 68158, Germany

## Abstract

**Motivation:**

In multi-cohort machine learning studies, it is critical to differentiate between effects that are reproducible across cohorts and those that are cohort-specific. Multi-task learning (MTL) is a machine learning approach that facilitates this differentiation through the simultaneous learning of prediction tasks across cohorts. Since multi-cohort data can often not be combined into a single storage solution, there would be the substantial utility of an MTL application for geographically distributed data sources.

**Results:**

Here, we describe the development of ‘dsMTL’, a computational framework for privacy-preserving, distributed multi-task machine learning that includes three supervised and one unsupervised algorithms. First, we derive the theoretical properties of these methods and the relevant machine learning workflows to ensure the validity of the software implementation. Second, we implement dsMTL as a library for the R programming language, building on the DataSHIELD platform that supports the federated analysis of sensitive individual-level data. Third, we demonstrate the applicability of dsMTL for comorbidity modeling in distributed data. We show that comorbidity modeling using dsMTL outperformed conventional, federated machine learning, as well as the aggregation of multiple models built on the distributed datasets individually. The application of dsMTL was computationally efficient and highly scalable when applied to moderate-size (*n* < 500), real expression data given the actual network latency.

**Availability and implementation:**

dsMTL is freely available at https://github.com/transbioZI/dsMTLBase (server-side package) and https://github.com/transbioZI/dsMTLClient (client-side package).

**Supplementary information:**

Supplementary data are available at *Bioinformatics* online.

## 1 Introduction

The biology of many human illnesses is encoded in a vast number of genetic, epigenetic, molecular, and cellular parameters. The ability of Machine Learning (ML) to jointly analyze such parameters and derive algorithms with potential clinical utility has fueled a massive interest in biomedical ML applications. One of the fundamental requirements for such ML algorithms to perform well is the availability of biological data at a large scale (Jahanshad *et al.*, 2013; Kochunov *et al.*, 2014; Schizophrenia Working Group of the Psychiatric Genomics Consortium, 2014). As data can often not be freely exchanged across institutions due to the need for the protection of an individual’s privacy, the utility of ‘bringing the algorithm to the data’ is becoming apparent. Technological solutions for this task have risen in popularity and exist in various forms.

One of the most straightforward approaches is federated ML, where algorithms are simultaneously learned at different institutions and optimized through a privacy-preserving exchange of parameters. In biomedicine, this approach has been applied in several important applications to prevent data leakage. For example, a federated solution based on the secure multi-party computation was proposed for whole-genome variant-querying (Akgun *et al.*, 2022), and the identification of disease-associated variants (Akgun *et al.*, 2021) by combing multiple distributed genomic data resources. Another work (Zolotareva *et al.*, 2021) explored the possibility of the federated learning approach for differential gene expression analysis of multiple resources and highlighted its utility with regard to its robustness against class imbalance. A recently developed ‘swarm-learning’ approach (Warnat-Herresthal *et al.*, 2021) introduced the blockchain technique in a distributed ML scenario in order to protect the security of data, and has been successfully applied for a large-scale analysis related to COVID-19. A detailed review of federated learning in biomedicine can be found in Rieke *et al.* (2020).

One commonality of most ML approaches, federated or not, is the assumption that all investigated observations (e.g. illness-affected individuals) represent the same underlying population (IID assumption). However, in multi-modal, biomedical data analysis, this is rarely the case, as biological and technological factors frequently induce modality-specific effects that are difficult to capture by federated ML. Multi-task Learning (MTL) can address this issue through the simultaneous learning of outcome (e.g. diagnosis) associated patterns across datasets with dataset-specific, as well as shared, effects. MTL has numerous exciting application areas, such as comorbidity modeling, and has already been applied successfully for, e.g. disease progression analysis (Zhou *et al.*, 2013).

**Fig. 1.**
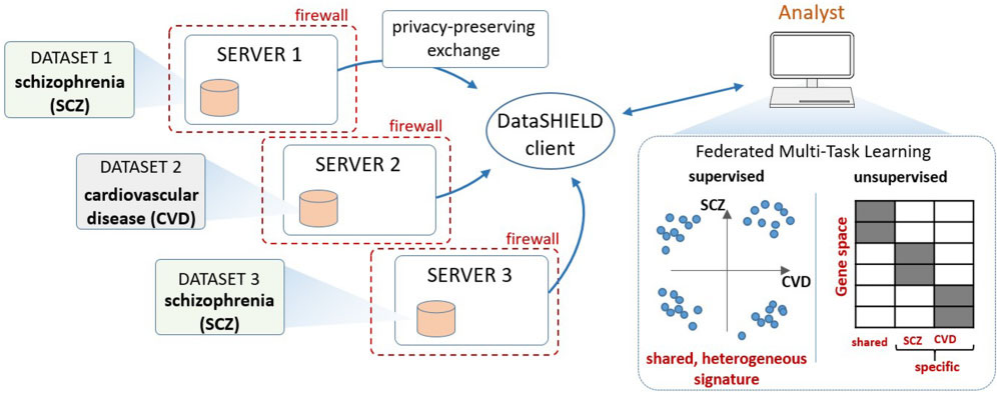
Schematic illustration of dsMTL using comorbidity modeling of schizophrenia and cardiovascular disease as an example. Multiple datasets stored at different institutions are used as a basis for federated MTL. dsMTL was developed in the DataSHIELD ecosystem, which provides functionality regarding data management, transmission and security. Data are analyzed behind a given institution’s firewall and only algorithm parameters that do not disclose personally identifiable information are exchanged across the network. dsMTL contains algorithms for supervised and unsupervised multi-task machine learning. The former aims at identifying shared, but potentially heterogeneous signatures across tasks (here, diagnostic classification for schizophrenia and cardiovascular disease). Unsupervised learning separates the original data into shared and cohort-specific components, and aims to reveal the corresponding outcome-associated biological profiles

Here, we describe the development of dsMTL (‘Federated Multi-Task Learning for DataSHIELD’), a package of the statistical software R, for Federated Multi-Task Learning (FeMTL) analysis (Fig. 1). dsMTL was developed for DataSHIELD (Gaye *et al.*, 2014), a platform supporting the federated analysis of sensitive individual-level data that remains stored behind the data owner’s firewall throughout analysis (Wilson *et al.*, 2017).

We explored the utility of the dsMTL algorithms for comorbidity analysis based on simulation data. Using data with variable degrees of cross-dataset heterogeneity aimed at quantifying the ability of the MTL algorithms to suitably characterize shared and specific biological signatures. We then showed the scalability of dsMTL methods for up to 20 servers compared to the federated ML methods. In addition, we analyzed actual RNA sequencing and microarray data in order to show that: (i) such analysis can be performed in an acceptable runtime using dsMTL given the real network latency, and (ii) federated MTL can capture more reproducible genes signatures compared to federated ML.

## 2 Materials and methods

### 2.1 Modeling

All methods of dsMTL share the identical form,

$$\underset{\theta }{\min}L\left(\theta \right)+\lambda S\left(\theta \right)+Cℵ\left(\theta \right)$$

where $L\left(\theta \right)$ is the data fitting term (or loss function), the major determinant of the solutions obtained from model training. Theta represents the parameters of the model. $ℵ\left(\theta \right)$ and S(θ) are the penalties of θ with the aim to incorporate the prior information. $S\left(\theta \right)$ is a non-smooth function and able to create sparsity, while ℵ(θ) is smooth, in order to stabilize the solution. λ and C are the hyper-parameters to control the strength of the penalties.

Using this framework, four federated MTL algorithms were derived, including three supervised and one unsupervised algorithms, that represent extensions of previously developed non-federated MTL implementations (Cao *et al*., 2019; Quintero *et al.*, 2020; Yang and Michailidis, 2016). When analyzing multi-cohort data, these methods can be seen as tools to differentiate between an effect shared among all cohorts and cohort-specific effects. Specifically, the dsMTL_L21 approach allows for cross-task regularization, building on the popular LASSO method, in order to identify outcome-associated signatures with a reduced number of features shared across tasks while remaining variability for each model to learn the respective disease-specific patterns. The non-federated version of this approach was previously applied to simultaneously predict multiple oncological outcomes using gene expression data (Xu et al., 2011). The dsMTL_trace approach constrains the coefficient vectors in a shared low-dimensional space during the training procedure while retaining a sufficient amount of variability for each coefficient vector to learn a given, cohort-specific pattern, resulting in improved generalizability of the models. In a non-federated implementation, this method has previously been used to predict the response to different drugs, and the identified models showed a high degree of interpretability in the context of the represented drug mechanism (Yuan *et al.*, 2016). dsMTL_Net incorporates a task-task network as the shared structure in order to improve biological interpretability. In a non-federated version, this technique has previously been used for the integrative analysis of heterogeneous cohorts (Cao *et al.*, 2018) and for predicting disease progression (Zhou *et al.*, 2011). The dsMTL_iNMF approach is an unsupervised, integrative non-negative matrix factorization method that aims at factorizing the cohorts’ data matrices into components, where one component is considered to be a superposition of a shared and a disease-specific structure. Such modeling has been applied to explore dependencies in multi-omics data for biomarker identification (Fujita *et al.*, 2018; Yang and Michailidis, 2016). In addition to the federated MTL methods, we also implemented a federated version of the conventional Lasso (dsLasso) (Tibshirani, 1996) in the dsMTL package due to its wide usage in biomedicine and as a benchmark for testing the performance of the federated MTL algorithms. The derivation of the mathematical properties, optimization procedure and machine learning workflows of each algorithm are summarized in the Supplementary Methods.

### 2.2 Efficiency

Most dsMTL methods aim at training an entire regularization tree. The determination of the λ sequence controls the tree’s growth and is essential for computational speed. The λ sequence should be accurately scaled to both capture the highest posterior and avoid overwhelming computations. Inspired by a previous study (Friedman *et al.*, 2010), we estimate the largest and smallest λ from the data by characterizing the optima of the objective using the first-order optimal condition and then interpolate the entire λ sequence on a log scale (see Supplementary Methods for more details). In addition, several options are provided to improve the speed of the algorithms by decreasing the precision of the results, i.e.: (i) the number of digits of parameters for transformation can be specified to reduce the network latency; (ii) several termination rules are provided, some of which are relaxed; (iii) the depth of the regularization tree can be shortened. More details can be found in the Supplementary Methods. Besides the efficiency of the federated ML/MTL methodology, the import/export of the large dataset is efficient in dsMTL (see the Supplementary Material for details).

### 2.3 Security

dsMTL was developed based on DataSHIELD (Wilson *et al.*, 2017), which provides comprehensive security mechanisms that are not specific to machine learning applications. These security mechanisms are summarized in the Supplementary Material and are inherited by dsMTL. In addition, we considered potential ML-specific privacy leaks, such as membership inference attacks (Hu *et al.*, 2021) and model inverse attacks (Fredrikson *et al.*, 2014). Inverse attacks aim at extracting the individual observation-level information from the models. Membership inference attempts to decide if an individual was included in a given training set using the model. All these techniques require a complete model for inference. Since MTL returns multiple matrices, returning an incomplete model could be one strategy against these attacks. For example, dsMTL_iNMF in dsMTL only returns the homogenous matrix (H), whereas the cohort-specific components ($V_{k},\;W_{k}$) never leave the server. For example, in a two-server scenario, one (H) out of five output matrices is transmitted between the client and the servers. With such an incomplete model, the inverse construction of the raw data matrix becomes difficult, and the risk of an inverse attack and membership inference is reduced. For most biomedical analyses, the H matrix is sufficient for subsequent studies. In addition, if the analyst was authorized to access the raw data of the server, the so-called ‘data key mechanism’ (see Supplementary Material) would allow the analyst to retrieve all component matrices. For supervised multi-task learning methods in dsMTL, all models have to be aggregated within the clients, and thus we suggest the data providers enable the option on the server that rejects a returned coefficient vector containing parameter numbers exceeding the number of subjects. In this way, the model is not saturated and more robust to an inverse attack.

### 2.4 Proof of concept with simulation and actual data

Simulation and actual data analysis were conducted to demonstrate the suitability of dsMTL methods to analyze heterogeneous cohorts, compared to federated ML methods and the ensemble of local models regarding the prediction performance, interpretability and computational speed. An overview of methodological aspects related to these analyses is detailed below. For an extensive methodological description, please see the Supplementary Material.

Two simulation studies were conducted to illustrate the utility of dsMTL for analyzing multi-cohort data with shared and cohort-specific signatures, for example in the case of comorbidity analysis. In case study 1, we considered a comorbidity scenario between two conditions that show changes in the same set of genes, albeit in potentially different directions (i.e. up- versus down-regulated). Here, we call these signatures ‘heterogeneous’. In contrast, ‘homogeneous’ signatures relate to the same features and signs across datasets. We aimed to capture this shared information using the supervised dsMTL method dsMTL_L21 and assessed its predictive performance as well as model interpretability. In case study 2, we assumed that the outcome-associated signature was the sum of the comorbidity signature and two disease-specific signatures. This mixed structure can be disentangled by the unsupervised dsMTL method dsMTL_iNMF. A detailed description of the simulated data can be found in the Supplementary Material.

In addition, a scalability analysis was conducted using simulation datasets to show the dependence of dsMTL’s efficiency on the number of servers. The efficiency was quantified as the communication cost, i.e. the number of network accesses, which has been recognized as the primary bottleneck of efficiency for federated applications (Li *et al.*, 2020). We scaled the number of participating servers from 1 to 20 and trained the dsMTL_L21 (federated MTL) as well as dsLasso (federated ML) models for each given number of servers. The number of network accesses was counted as the basis for the comparison. Furthermore, we varied the number of subjects, in order to explore the dependency of the communication cost on the sample size.

Two studies were performed on real gene expression datasets, in order to investigate the efficiency and interpretability of dsMTL. For supervised analysis, a three-server MTL analysis was conducted to assess the running speed. For the unsupervised analysis, we simultaneously investigated data from patients with schizophrenia and bipolar disorder, and quantified the running speed on two actual servers. In order to explore the biological plausibility of the identified gene signatures, we performed a pathway enrichment analysis. Furthermore, in order to demonstrate the reproducibility of the gene signatures found by dsMTL, the overlap of the signatures found by dsMTL using different datasets for training was investigated. For this analysis, the ensemble of local models was used as the baseline. A detailed description of the preprocessing, as well as the analysis of real gene expression data can be found in the Supplementary Material.

## 3 Results

### 3.1 Simulation data analysis

#### 3.1.1 Case study 1—distributed MTL for identification of heterogeneous target signatures

**Fig. 2.**
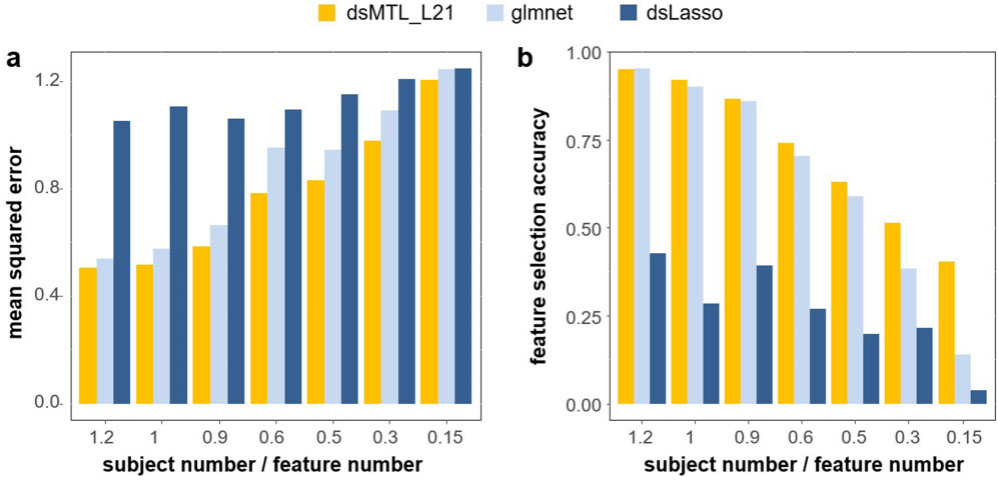
Analysis of ‘heterogeneous’ signatures of continuous outcomes in simulated data stored on three servers. The figure shows the: (**a**) prediction accuracy expressed as the mean squared error and (**b**) the feature selection accuracy for different subject/feature number ratios. The respective values were averaged across the three servers, and across 100 repetitions, in order to account for the effect of sampling variability

With the aim to identify ‘heterogeneous’ signatures, we compared the performance of dsMTL_L21, dsLasso and the bagging of glmnet models. As part of this, we explored the sensitivity of these methods to different sample sizes (*n*) relative to the gene number (*p*). Figure 2 shows the resulting prediction performance and gene selection accuracy, each averaged over 100 repetitions. dsLasso showed the worst prediction performance in this heterogeneous setting, and dsMTL_L21 slightly outperformed the aggregation of local models (glmnet). Similarly, the gene selection accuracy of dsLasso was inferior to that of dsMTL_L21 and glmnet-bagging, which showed similar performance when the sample size is sufficiently large, e.g. the number of subjects approximately equal to the number of genes (*n*/*p* ∼1). However, with a decreasing *n*/*p* ratio, dsMTL_L21 showed an increasing superiority over the other methods, especially for *n*/*p* = 0.15, where the gene selection accuracy of dsMTL_L21 was more than 2.8 times higher than that of the bagging technique.

#### 3.1.2 Case study 2—distributed iNMF for disentangling shared and cohort-specific signatures

**Fig. 3.**
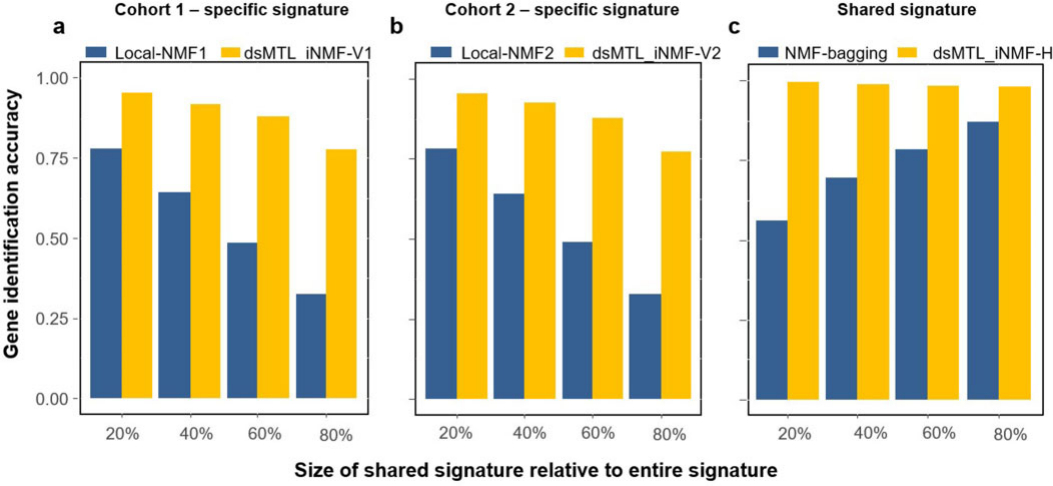
The gene identification accuracy for shared and specific signatures using simulated data. (**a**) The identification accuracy of cohort-specific genes for cohort 1. (**b**) The identification accuracy of cohort-specific genes for cohort 2. (**c**) The identification accuracy of genes comprised in the shared signature. Local-NMF1 and Local-NMF2 were the cohort-specific gene sets identified by local NMF, which were combined into ‘NMF-bagging’ for the shared gene set. dsMTL_iNMF-H was the predicted shared gene set using dsMTL_iNMF. dsMTL_iNMF-V1 and dsMTL_iNMF-V2 were the predicted cohort-specific gene sets identified using dsMTL_iNMF. The proportion of genes harbored by the shared signature varied from 20% to 80%, illustrating the impact of the heterogeneity severity. The model was trained using rank = 4 as the model parameter. The results for a broader spectrum of rank choices can be found in Supplementary Figure S5, illustrating that the superior performance of dsMTL_iNMF was not due to the choice of ranks

Figure 3 shows the performance of distributed and aggregated local NMF methods for disentangling shared and cohort-specific signatures from multi-cohort data, given different ‘severities’ of the signature heterogeneity. For both types of signatures, dsMTL_iNMF outperformed the ensemble of local NMF models for any heterogeneity severity setting. Notably, even with increasing heterogeneity, the accuracy of dsMTL_iNMF to capture shared genes remained stable at approximately 100%, illustrating the robustness of dsMTL_iNMF against the heterogeneity’s severity shown in Figure 3c. In contrast, for the ensemble of local NMF, the gene selection accuracy of the shared signature continuously decreased to approximately 50% (20% of outcome-associated genes were shared among cohorts), while the gene selection accuracy of cohort-specific signatures continuously increased to 75% (20% of outcome-associated genes were shared among cohorts) as shown in Figure 3a and b.

#### 3.1.3 Scalability analysis

**Fig. 4.**
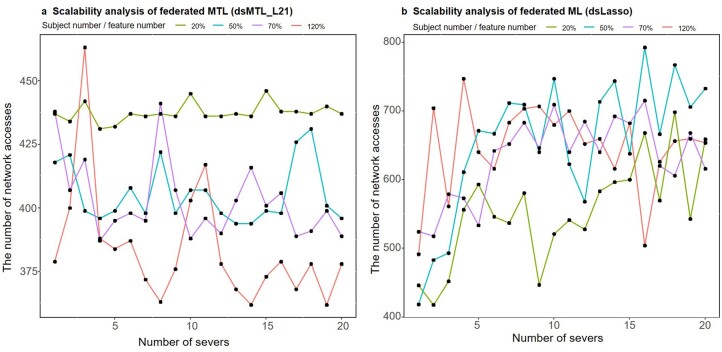
Scalability analysis for up to 20 servers. (**a**) The result of the dsMTL_L21 method. (**b**) The result of the dsLasso method. Both panels show the communication cost (e.g. the number of network accesses) with an increasing number of servers, and for different subject numbers by feature number ratios

Figure 4 shows the dependency of the communication cost, i.e. the number of network accesses, on the number of servers and subjects included in the analysis. An increasing communication cost of dsLasso (federated ML) was observed with an increasing number of servers, as shown in Figure 4b. However, for dsMTL_L21 (federated MTL), the communication cost was not dependent on the number of servers (Fig. 4a). This might be due to the fact that the gradient information, a major determining factor of the ML/MTL solution, can be calculated locally on each server for the federated MTL algorithm but needs information aggregation via the internet for the federated ML algorithm. Moreover, Figure 4a shows that for federated MTL, the communication cost decreased with an increasing subject number. Such a clear pattern was not found for federated ML, as shown in Figure 4b. This may suggest that a larger sample size might help to reduce the communication cost of the federated MTL algorithm.

### 3.2 Actual data analysis

#### 3.2.1 Computational speed analysis and biological enrichment

**Supervised dsMTL.** We first explored the efficiency of dsMTL using real molecular data of patients with schizophrenia, given the actual latency of a distributed network, and then investigated the biological relevance of the identified gene signatures. The efficiency was quantified for a three-server scenario (see Supplementary Table S3, two servers at the Central Institute of Mental Health, Mannheim; one server at BioQuant, Heidelberg University). We analyzed four case-control gene expression datasets of patients with schizophrenia and controls (median *n* = 80; 8013 genes). Table 1 shows the comparison between dsLasso and mean-regularized dsMTL_Net, which were trained (cross-validation + training) and tested in approximately 8 min and 10 min, respectively. The time-difference might be due to the high heterogeneity of the brain gene expression that can be disentangled by dsMTL_Net but that is ignored by dsLasso. Thus, a larger number of iterations were used by dsMTL_Net. To demonstrate the cross-cohort prediction performance of dsMTL_Net and dsLasso, we predicted the models on an independent brain expression dataset (HBCC). As shown in Table 1, the prediction accuracy of dsMTL_Net (misclassification rate: 0.29) was slightly higher than that of dsLasso (0.34), consistent with our previous study (Cao *et al.*, 2018). To explore biological interpretability and cross-tissue reproducibility, respectively, we performed pathway-related enrichment analysis of the top-ranked gene signatures and predicted the signatures in data from blood samples of patients with schizophrenia. The pathway enrichment analysis yielded 12 significant pathways, including pathways related to ion homeostasis, including ‘zinc ion homeostasis’ (*FDR *=* *0.0015) and ‘response to zinc ion’ (*FDR *=* *0.016), which have previously been associated with psychosis (Petrilli *et al.*, 2017). The complete list of significant pathways can be found in the Supplementary Material. Pathway enrichment analysis was based on the top 200 genes identified by dsMTL_Net, and strong overlap of pathways were observed when the top 300 (12/12 pathways) or 100 (7/12 pathways) genes were used, respectively. The cross-tissue prediction in data from blood samples of 859 subjects showed that patients could be significantly differentiated from controls (*t *=* *2.7, *P *=* *0.0055), suggesting that the brain-derived signatures were partly mirrored in blood samples.

**Table 1.** Performance of dsML/MTL models on real data in real network

	dsLasso	dsMTL_Net
Misclassification rate	0.34	0.29
Time consumed		
5-fold CV	7.5 min	7.3 min
Training	1.7 min	2.9 min
Number of network accesses for training	70	137
Non-zero coefficients	38	173

**Unsupervised dsMTL.** The cohorts and server information is shown in Supplementary Table S4. It took 34.9 min (1003 network accesses) to train a dsMTL_iNMF model with five random initializations (∼7 min for each initialization). The factorization rank *k* = 4 was selected as the optimal parameter. In Supplementary Figure S4, the objective curve shows that the training time was sufficient for model convergence. In this analysis, a shared signature comprising 1971 genes between SCZ and BIP was identified, while two disease-specific signatures containing 60 genes for SCZ and 1341 genes for BIP, respectively, were found. These genes were enriched for biologically plausible pathways. For example, for the schizophrenia-specific signature, the enriched pathways included ‘L1 cell adhesion molecule interactions (Kurumaji *et al.*, 2001)’, ‘chaperone mediated autophagy (Schneider *et al.*, 2016)’ and other relevant processes. The pathways linked to bipolar-specific signatures included several terms related to neuronal and synaptic processes, like ‘transmission across chemical synapses’ or ‘neurexins and neuroligins’, which have been related to psychotic disorders and in particular bipolar disorders (Cuttler *et al.*, 2021).

#### 3.2.2 Reproducibility analysis of gene signature identification

Table 2 shows that the gene signatures found by dsMTL_Net were more reproducible in independent data cohorts than the ones found by the glmnet-bagging method. As indicated in Table 2, four datasets were categorized into two distinct dataset groups, and the gene signatures were compared across these groups. For glmnet-bagging, on an average, around nine gene signatures were reproducibly found in the two distinct dataset groups. The number of overlapping genes with consistent association direction was slightly lower (8 on an average). However, for dsMTL_Net, on an average 13 genes were found simultaneously in the two dataset groups, and 12 genes on an average demonstrated the same direction. This showed that federated MTL had a stronger ability to identify reproducible gene signatures among heterogeneous datasets. To explore the cross-cohort predictive ability of these gene signatures, we predicted the model, which was trained on a given dataset group, in the respectively other. The average prediction accuracy was around 0.67 for both methods.

**Table 2.** The result of reproducibility analysis of signature identification

	Methods	Criteria	Experiment 1		Experiment 2		Experiment 3	
Datasets split			$\left\{a,\;b\right\}$	$\left\{c,\;d\right\}$	$\left\{a,\;c\right\}$	$\left\{d,\;b\right\}$	$\left\{a,\;d\right\}$	$\left\{c,\;b\right\}$
Validation of selected genes on independent cohorts	dsMTL_Net	Overlapping genes	11.68		11.78		14.81	
		Consistent direction	10.28		11.24		14.69	
	glmnet-bagging	Overlapping genes	7		6.75		12.89	
		Consistent direction	6		6.57		11.89	

*Note:* Four datasets were split into two distinct dataset groups. The model was trained on each dataset group individually. Then the top selected genes (200) from both models were compared to assess the signature reproducibility. The ‘overlapping genes’ referred to the gene signatures identified in all dataset groups. The ‘consistent direction’ referred to the overlapping genes demonstrating a consistent direction of outcome-associations. To address sampling variability, the analysis was repeated 100 times and results were averaged across repetitions. The notation of datasets is a: GSE53987, b: GSE21138, c: GSE35977, d: HBCC.

## 4 Discussion

We here present dsMTL—a secure, federated multi-task learning package for the programming language R, building on DataSHIELD as an ecosystem for privacy-preserving and distributed analysis. Multi-task learning allows the investigation of research questions that are difficult to address using conventional ML, such as the identification of heterogeneous, albeit related, signatures across datasets. The implementation of a privacy-preserving framework for the distributed application of MTL is an essential requirement for the large-scale adoption of MTL. Using such a distributed server setup, we demonstrate the applicability and utility of dsMTL to identify biomarker signatures in different settings. For applications where the target biomarker signatures are different, but relate to an overlapping set of features (explored here as the ‘heterogeneous’ case), conventional machine learning would not be a meaningful algorithm choice. We show that MTL is able to identify the target signatures with high confidence and may thus be a reasonable choice for a diverse set of interesting analyses. As mentioned above, a particularly noteworthy application is comorbidity modeling, where the target signatures index the shared (although potentially heterogeneously manifested) biology of multiple, clinically comorbid conditions. Such analyses could potentially be a powerful, machine learning-based extension of comorbidity modeling approaches based on univariate statistics that have already been very useful for characterizing the shared biology of comorbid illness (Lichtenstein *et al.*, 2009). Meanwhile, we showed the communication cost of federated MTL was independent of the number of servers, highlighting the scalability of dsMTL. Surprisingly, we found that an increasing subject number reduces the communication cost for federated MTL methods. This might be due to the fact that the network communication in federated MTL is aimed at identifying the shared effect among tasks, and such effect would be more efficiently identified in a large sample. In the actual data analysis, we showed that federated MTL was able to capture reproducible and biologically plausible gene signatures. Despite this reproducibility, the number of reproduced genes (e.g. overlapping genes: 13 on an average) was comparatively low, likely a reflection of the pronounced heterogeneity of the brain tissue expression datasets. We show that unsupervised MTL could disentangle the shared from cohort-specific effects, demonstrating its potential utility for comorbidity analysis. Other applications for this method include the analysis of biological patterns shared across or specific to diverse single-cell measurements (Welch *et al.*, 2019).

We selected R as the major implementation language to make dsMTL able to cooperate with other DataSHIELD-based or CRAN packages, based on the concept of a ‘freely composing script’. This means the dsMTL workflow was formed as a composition of dsMTL, DataSHIELD, and local R commands (e.g. R base functions, customer-defined functions and CRAN packages) into a script, such that the geo-distribution of datasets and the federated computation are transparent to users. This concept is similar to that of the ‘freely composing apps’ used in a recently presented federated ML application (Matschinske et al., 2021), which allows flexible scheduling of functions in the form of apps and improves the federated data analysis flexibility for users. Considering there are thousands of well-established packages already on CRAN for different analysis aims, this will significantly broaden the potential applicability of dsMTL. All DataSHIELD-based packages are listed here (Consotia, 2019).

Interesting future developments of the dsMTL approach could include the implementation of asynchronous gradient descent, which provides a probabilistically approximate solution but faster convergence (Xie *et al.*, 2017; Zhang and Liu, 2020). Furthermore, the integration of other popular systems for ML, such as tensorflow (Dahl *et al.*, 2018), for which interfaces with the R language already exist, would provide valuable additions to the DataSHIELD system. In addition, the re-implementation of gradient calculation functions on the server side into a low-level language, i.e. C++ would significantly improve the efficiency.

Finally, a noteworthy consideration is an architecture underlying the distributed data infrastructure. DataSHIELD builds on a centralized (‘client-server’) architecture and each data provider needs to install a well-configured data warehouse. Such infrastructure is suitable for long-term collaboration scenarios and large consortia projects that conduct a broad spectrum of complex analyses requiring high flexibility. However, in other scenarios that require more temporary and easy-compute collaboration setups, a server-free or decentralized architecture (Warnat-Herresthal *et al.*, 2020) might be more suitable, because the cost of data provider for participating is low.

In conclusion, the dsMTL library for R programming provides an easy-to-use framework for privacy-preserving, federated analysis of geographically distributed heterogeneous datasets. Due to its ability to disentangle shared and cohort-specific effects across these datasets, dsMTL has numerous interesting application areas, including comorbidity modeling and translational research focused on the simultaneous prediction of different outcomes across datasets.

### Consortium authors: The COMMITMENT Consortium

Emanuel Schwarz^1,^*, [alphabetical order starts] Dag Alnæs^2^, Ole A. Andreassen^2^, Han Cao^1^, Junfang Chen^1^, Franziska Degenhardt^3,4^, Daria Doncevic^5^, Dominic Dwyer^6^, Roland Eils^5,7^, Jeanette Erdmann^8^, Carl Herrmann^5^, Martin Hofmann-Apitius^9^, Nikolaos Koutsouleris^6,10^, Alpha T. Kodamullil^9^, Adyasha Khuntia^6^, Sören Mucha^8^, Markus M. Nöthen^3,11^, Riya Paul^6^, Mads L. Pedersen^12^, Heribert Schunkert^13^, Heike Tost^1^, Lars T. Westlye^2,12^, Youcheng Zhang^5^, [alphabetical order ends], Andreas Meyer-Lindenberg^1,^*

^1^Department of Psychiatry and Psychotherapy, Central Institute of Mental Health, Medical Faculty Mannheim, Heidelberg University, Mannheim, Germany, ^2^Norwegian Centre for Mental Disorders Research (NORMENT), Division of Mental Health and Addiction, Oslo University Hospital and Institute of Clinical Medicine, University of Oslo, Oslo, Norway, ^3^Institute of Human Genetics, University of Bonn, School of Medicine & University Hospital Bonn, Bonn, Germany, ^4^Department of Child and Adolescent Psychiatry, Psychosomatics and Psychotherapy, University Hospital Essen, University of Duisburg-Essen, Duisburg, Germany, ^5^Health Data Science Unit, Medical Faculty Heidelberg and BioQuant, Heidelberg 69120, Germany, ^6^Department of Psychiatry and Psychotherapy, Section for Neurodiagnostic Applications, Ludwig-Maximilian University, Munich 80638, Germany, ^7^Center for Digital Health, Berlin Institute of Health and Charité, Berlin, 10117, Germany, ^8^Institute for Cardiogenetics, University of Lübeck, DZHK (German Research Centre for Cardiovascular Research), Partner Site Hamburg/Lübeck/Kiel, and University Heart Center Lübeck, Lübeck, Germany, ^9^Fraunhofer Institute for Algorithms and Scientific Computing (SCAI), Sankt Augustin 53754, Germany, ^10^Max-Planck Institute of Psychiatry, Munich, Germany, ^11^Department of Genomics, Life & Brain Center, University of Bonn, Bonn, Germany, ^12^Department of Psychology, University of Oslo, Oslo, Norway, ^13^Department of Cardiology, Deutsches Herzzentrum München, Technische Universität München, Munich Heart Alliance (DZHK), Germany.

## Supplementary Material

btac616_Supplementary_Data

## Data Availability

The data underlying this article are available in the GEO (GSE53987, GSE21138 and GSE35977) and the dbGaP (ID: phs000979.v3.p2) repositories.
