# Supplementary material for: dsMTL: a computational framework for privacy-preserving, distributed multi-task machine learning
Source: Bioinformatics. 2022 Sep 8;38(21):4919–26. doi: 10.1093/bioinformatics/btac616 (PMC9620828; doi:10.1093/bioinformatics/btac616)
Supplement: btac616_Supplementary_Data [file btac616_supplementary_data.zip › Cao_etal_Bioinfo_supplMaterials_clean_copy_final.docx]

# Outline

1, dsMTL framework

- Notions of variables
- The distributed unsupervised method in dsMTL
  - Distributed variables update
  - Algorithm
- Distributed supervised methods in dsMTL
  - Models
- Distributed optimization framework
  - Solving sub-problem in each iteration
  - Line search
  - Federated computation
  - Accelerated algorithms

2, Introduction of DataSHIELD

3, Data key mechanism

4, Generation of RNA-seq count data for case study 2

5, Pre-processing of actual expression datasets

- Microarray data for supervised dsMTL
- RNA-seq data for unsupervised dsMTL

6, Simulation data analysis

- Two case studies
- Scalability analysis up to 20 servers

7, Actual data analysis

- Computational speed analysis
- Cross-tissue prediction and pathway enrichment analysis
- Reproducibility analysis of gene signatures

# dsMTL framework

In dsMTL, we included four federated multi-task (FeMTL) and one machine learning (FeML) methods covering supervised and unsupervised learning procedures. These methods are inherited from non-federated MTL packages, i.e., RMTL^1^ for supervised and ShinyButchR^2^ for unsupervised approaches. These methodologies were selected for the following reasons. First, the high-dimensional biological data are the primary envisaged training data types for dsMTL, and we are particularly interested in the regularization-based MTL. Second, for biomedical analysis, the interpretability of a given model is a critical criterium for model selection. Therefore, we were particularly interested in the feature selection accuracy of the machine learning algorithms, and thus the introduction of sparsity in dsMTL methods.

All models followed the consistent formulation,

$\min_{\theta} \mathcal{L}\left( \theta\right)+\lambda S\left( \theta\right)+C\aleph\left( \theta\right)$ (1)

$\mathcal{L}\left( \theta\right)$ was the data fitting term (or loss function), the major determinant of the solutions of the model training. $\aleph\left( \theta\right)$ and $S(\theta)$ were the regularization/penalty terms with the aim to incorporate the prior information and prevent overfitting. $\aleph\left( \theta\right)$ was a non-smooth function for creating the sparsity, while $S(\theta)$ was smooth with the ability to stabilize the solution. $\lambda$ and $C$ were the hyper-parameters to control the strength of the penalty, $\lambda$ was learned from cross-validation (CV) and $C$ was the constant.

There are three loss functions in dsMTL, achieving the tasks of regression, classification and matrix factorization. They are summarized in **Supplementary Table 1**.

**Termination rules.** Four termination rules were included in dsMTL to determine whether the optimization converges. The first three rules were applied to all methods in dsMTL, while the last was newly designed for matrix factorization. The first rule checked whether the current objective value was close enough to 0. The second rule investigates the last two objective values and checks whether the decrement was close enough to 0. The third rule allowed the optimization to be performed for a certain maximum number of iterations. The last rule specific to matrix factorization is described in the next section.

## Notions of Variables

The variables used in this section are defined here.

| $\theta$ | The parameters’ set for estimation |
| --- | --- |
| $k$ | The index of the task |
| $t$ | The number of tasks |
| $n$ | The number of subjects for a dataset |
| $n_{k}$ | The number of subjects for task k |
| $\left\{ x,y \right\}$ | The design matrix (x) and the relevant outcomes (y) |
| $\left\{ X_{k}, Y_{k} \right\}$ | The design matrix and the relevant outcomes for task k |
| $\left\{ X_{i}^{(k)},Y_{i}^{(k)} \right\}$ | The data and outcome of the subject i and task k |
| $X_{,j}^{(k)}$ | The feature column j of the task matrix $X_{k}$ |
| $\left\{ X_{i}^{(k)},Y_{i}^{(k)} \right\}$ | The data and outcome of the subject i and task k |
| $C$ | The hyper-parameter that need be determined by user |
| $W_{k}$ | The parameter matrix W for task k |
| $W_{i,}^{(k)}$ | The row vector of $W_{k}$ |

## Federated unsupervised method in dsMTL

To discover the hidden structure in heterogeneous, high-dimensional biological data, we integrated the integrative matrix factorization method^3^ (iNMF) in our distributed learning framework, called dsMTL_iNMF. The major concept of dsMTL_iNMF is shown in **Supplementary Figure 1**, where the cohort matrices on three servers can be factorized simultaneously with the shared component matrix ($H$) and cohort-specific component matrices($V$, $W$). The objective function was

$$\min_{\begin{aligned} H, \\ W_{1}\ldots,W_{t}, \\ V_{1}\ldots,V_{t} \\ \end{aligned}} \sum_{k=1}^{t} {||X_{k}-(H+V_{k})W_{k}||}_{F}^{2}+\lambda\sum_{k=1}^{t} {||V_{k}W_{k}||}_{F}^{2}+\lambda_{s}\sum_{k=1}^{t} {|W_{k}|}_{1}$$

The robustness of the model was due to the decoupled setting of $H$ and $V_{k}$, where $H$ was to capture the shared information across cohorts and $V_{k}$ was to capture the cohort-specific information. To integrate more information into the shared component H, the magnitude of the cohort-specific component was penalized$\aleph\left( . \right)=\sum_{k=1}^{t} {||V_{k}W_{k}||}_{F}^{2}$. The sparse term $S\left( . \right)=\sum_{k=1}^{t} {|W_{k}|}_{1}$ was used to remove the redundant coefficients from the component matrices.

### Distributed variables update

$W_{k_{ij}}\leftarrow W_{ij}\frac{\left( {(H+V_{k})}^{T}X_{k} \right)_{i,j}}{\left( (H^{T}H+H{V_{k}}^{T}+H^{T}V_{k}+(1+\lambda){V_{k}}^{T}V_{k})W_{k} \right)_{i,j}+\lambda_{s}}$ (2)

$V_{k_{ij}}\leftarrow V_{ij}\frac{\left( X_{k}W_{k}^{T} \right)_{i,j}}{\left( HW_{k}W_{k}^{T}+(1+\lambda)V_{k}W_{k}W_{k}^{T} \right)_{i,j}+\lambda_{s}}$ (3)

$H_{ij}\leftarrow H_{ij}\left( \frac{X_{1}W_{1}^{T}+\ldots+X_{t}W_{t}^{T}}{\left( H+V_{1} \right)W_{1}W_{1}^{T}+\ldots+\left( H+V_{t} \right)W_{t}W_{t}^{T}} \right)_{i,j}$ (4)

The variables were updated for non-federated applications as demonstrated in formulas (2) to (4). In the federated scenario, the cohort-specific variables $W_{k}$ and $V_{k}$ were updated on server k using the local data as formulas (2) and (3). The shared matrix $H$ was updated on the client after receiving summary data (see **Supplementary Figure 1**) from all servers, where these were not-disclosed and calculated behind a given institution’s firewall. The distributed update of $H$ was

$H_{ij}\leftarrow H_{ij}\left( \frac{{server}_{1}(X_{1}W_{1}^{T})+\ldots+{{server}_{t}(X}_{t}W_{t}^{T})}{{server}_{1}(\left( H+V_{1} \right)W_{1}W_{1}^{T})+\ldots+{server}_{t}(\left( H+V_{t} \right)W_{t}W_{t}^{T})} \right)_{i,j}$ (5)

After the aggregation, the client updates $H$ and a new iteration begins. The communications between the client and the servers are illustrated in **Supplementary Figure 1**.

### Algorithms

**Distributed solver**

For the privacy-preserving purpose, only the shared matrix $H$ was returned. The distributed solver of dsMTL_iNMF is shown in Algorithm 1.

**Algorithm 1**, Solver of distributed iNMF in dsMTL

**Input**: $\lambda>0$, $\lambda_{s}>0$, $\mathrm{maxIter}>0$, $H$, $W_{1}\ldots,W_{t}$, $V\ldots,V_{t}$

**Output**:$H$

1: **for** $i=1$ to $\mathrm{maxIter}$ **do**

2: Update $H$ according to (5) on client

3: Send $H$ to all servers

3: Update $W_{1}\ldots,W_{t}$ according to (2) on server $1,\ldots,k$

4: Update $V_{1}\ldots,V_{t}$ according to (3) on server $1,\ldots,k$

5: Send summary statistics back client according to (5)

6: If termination rule satisfied, **return**

7: **end for**

**Termination rules**.

For dsMTL_iNMF, we provided an additional termination rule developed in the ButchR package^2^ to determine the convergence of the algorithm as shown in **Supplementary Figure 2**. In this method, each of the samples was assigned to a hidden factor (clustering membership) by $j=\arg\max_{j} |H_{i,j}|$ at every iteration. The convergence was determined when the assignments of samples remained unchanged. By default, if the samples were assigned to the same hidden factors consistently for over 10 iterations, the memberships were seen as stable, and the algorithm stopped. The rationale behind this procedure is that in order to maximize the power of clustering, the variance of the determined memberships must be small. Therefore, the proposed rule terminates the algorithm when the samples find stable memberships, such that the clustering can make a stable decision.

**Distributed model training.**

In the default setting, Algorithm 1 was performed with 10 random initial points to approximately sample the distribution of the local optima considering the non-convex nature of the problem. The initialization of these component matrices were uniformly sampled from $[0, 2]$. For each initialization, Algorithm 1 was performed. A set of shared matrices were returned as the final results for subsequent analysis

**Algorithm 2** Training procedure of iNMF in dsMTL

**Input**:$\lambda>0$, $\lambda_{s}>0$, $\mathrm{maxIter}>0$, rank, nInitialization, $\left\{ X_{1}, \ldots X_{k}, \ldots X_{t} \right\}$

**Output**:$H_{1}$, $H_{2}$,…

1: **for** $i=$ 1 to nInitialization **do**

2: Initialize $H_{i}\sim U_{n\times rank}(0,1)$, for each k, $V_{k}\sim U_{n\times rank}(0,1)$, $W_{k}\sim U_{rank\times p_{k}}(0,1)$

3: $H_{i}=$**Algorithm 1** $\boldsymbol{(}\lambda=\lambda$,$\lambda_{s}=\lambda_{s}$, $H=H_{i}$, $\left\{ W_{1}, \ldots,W_{t} \right\}$, $\left\{ V_{1}, \ldots,V_{t} \right\}$)

4: **end for**

## Federated supervised methods in dsMTL

We included one machine-learning (ML) and three multi-task learning (MTL) algorithms into supervised methods of dsMTL. In the federated scenario, the ML model was trained by averaging the summary statistics from geo-distributed cohorts with the synchronous communication, which leads to a model equivalent to the standalone ML model training on the concatenated cohorts. MTL, in the federated scenario, exchanges a small amount of information by regularization, such that the commonality of multi-cohort models was reinforced, but the cohort-specific element remained unchanged. In dsMTL, dsLasso was included as the federated ML variant of Lasso^4^. The federated multi-task learning methods were adapted from our previously published package RMTL^1^. These methods adopted various strategies of cross-cohort regularization to select joint features, explore the low-rank structure and incorporate the network structure in multiple tasks.

In the next sections, we introduce the theoretical derivations of each method to form the federated algorithm. Then a federated optimization framework is derived that was applied to all supervised methods. At the end, we show an executable algorithm for solving the objective and training the model.

### Models

For each method, the objective function is first introduced as the major problem to solve. Second, we derive the subgradient of the objective to characterize the properties of the optima. Third, since all models are sparse, we aimed to solve the entire regularization tree^5^ with a given positive $\lambda$sequence in decreasing order. The$\lambda_{max}$, the estimate of the largest$\lambda$in the$\lambda$sequence, was derived from the subgradient, such that $\lambda_{max}$ was the smallest $\lambda$ guaranteeing the existence of 0 optima. Last, to solve the non-smooth objective efficiently, the proximal mapping was applied, and the proximal point estimator was derived as the solution to the iteration-level sub-problem (see next section).

***Lasso (dsLasso)***

Objective function:

$$\min_{w} \frac{1}{n}\sum_{i=1}^{n} \mathcal{L}\left( w;X,Y \right)+\lambda\left| w \right|+C{||w||}_{2}^{2}$$

Subgradient:

$$\partial_{w}=\nabla_{w}\mathcal{L}+2Cw+\lambda\frac{w}{|w|}$$

Estimated $\lambda_{max}$:

$$\lambda_{max}=\left\{ \begin{aligned} \begin{matrix} \frac{1}{n}\max_{j} |X_{,j}^{T}Y| & for least square loss \end{matrix} \\ \begin{matrix} \frac{1}{2n}\max_{j} |X_{,j}^{T}Y| & for logit loss \end{matrix} \end{aligned} \right.$$

Proximal point estimation:

$$\underset{f=\frac{\lambda\left| x \right|}{L}}{\mathrm{prox}} \left( w \right)=\mathrm{sign}(w)max\left\{ w-\lambda,0 \right\}$$

The Lasso model^4^ aimed to learn a sparse parameter vector $w$. $\lambda$ was identified by cross-validation. ${||w||}_{2}^{2}$ was used to stabilize the solution and incorporate the correlated features. $C$ was selected by the user.

***MTL with Feature selection (dsMTL_L21)***

Objective function

$$\min_{W} \sum_{k=1}^{t} \sum_{i=1}^{n_{k}} \frac{1}{n_{k}}\mathcal{L}\left( W_{,k};X_{i}^{(k)},Y_{i}^{(k)} \right)+\lambda\sum_{j=1}^{p} \sqrt{{||W_{j,}||}_{2}^{2}}+C{||W||}_{2}^{2}$$

Subgradient:

$\partial_{W_{j,}}=\nabla_{W_{j,}}\mathcal{L}+2CW_{j,}+\lambda v$, $v=\left\{ x\in R^{t}: {||x||}_{2}\leq1 \right\}$

Estimated $\lambda_{max}$:

$$\lambda_{max}=\left\{ \begin{aligned} \begin{matrix} \max_{j} \sqrt{\sum_{k=1}^{t} \left( \frac{<X_{,j}^{(k)},Y^{(k)}>}{n_{k}} \right)^{2}} & for least square loss \end{matrix} \\ \begin{matrix} \max_{j} \sqrt{\sum_{k=1}^{t} \left( \frac{<X_{,j}^{(k)},Y^{(k)}>}{2n_{k}} \right)^{2}} & for logit loss \end{matrix} \end{aligned} \right.$$

Proximal point estimation:

$$\underset{f=\frac{\lambda{||x||}_{2}}{L}}{\mathrm{prox}} \left( w \right)=\left( 1-\frac{\lambda}{max\left\{ \left| \left| w \right| \right|_{2},\lambda\right\}} \right)w$$

The method^6^ aimed to find a model with the same set of features. For this, $\sum_{j=1}^{p} \sqrt{{||W_{j,}||}_{2}^{2}}$ was used to penalize the magnitudes of the coefficients of a given feature across the datasets. The $W=p\times t$ was the solution matrix of $t$ tasks and $p$ features.

***MTL with low-rank structure(dsMTL_Trace)***

Objective function:

$$\min_{W} \sum_{k=1}^{t} \sum_{i=1}^{n_{k}} \frac{1}{n_{k}}\mathcal{L}\left( W_{,k};X_{i}^{(k)},Y_{i}^{(k)} \right)++\lambda{|\left| W \right||}_{*}+C{||W||}_{2}^{2}$$

Subgradient:

$$\partial_{W}=\nabla_{W}\mathcal{L}+2CW+\lambda\partial{|\left| W \right||}_{*}$$

Estimated $\lambda_{max}$:

$$\lambda_{max}=\left\{ \begin{aligned} \begin{matrix} \max_{j} \sigma_{1}(\left[ \frac{{X^{\left( 1 \right)}}^{T}Y^{\left( 1 \right)}}{n_{1}}, \ldots,\frac{{X^{\left( t \right)}}^{T}Y^{\left( t \right)}}{n_{t}} \right]) & for least square loss \end{matrix} \\ \begin{matrix} \max_{j} \sigma_{1}(\left[ \frac{{X^{\left( 1 \right)}}^{T}Y^{\left( 1 \right)}}{2n_{1}}, \ldots,\frac{{X^{\left( t \right)}}^{T}Y^{\left( t \right)}}{2n_{t}} \right]) & for logit loss \end{matrix} \end{aligned} \right.$$

Where $\sigma_{1}(A)$is the largest singular value of matrix A

Proximal point estimation:

$\underset{f=\frac{\lambda{||x||}_{*}}{L}}{\mathrm{prox}} \left( W \right)=U\times I_{max\left\{ \sigma-\lambda,0 \right\}}\times V$,

where $W=U\Sigma V$, $\sigma$ is the diagonal vector of $\Sigma$

The method^7^ aimed to identify the coefficient vectors of multiple cohorts existing in the compressed low-dimensional space. For this, the trace norm of the coefficient matrix was used to compress the models’ space.

***MTL with network structure(dsMTL_Net)***

Objective function

$$\min_{W} \sum_{k=1}^{t} \sum_{i=1}^{n_{k}} \frac{1}{n_{k}}\mathcal{L}\left( W_{,k};X_{i}^{(k)},Y_{i}^{(k)} \right)+\lambda{|\left| W \right||}_{1}+C{|\left| GW \right||}_{2}^{2}$$

Subgradient:

$$\partial_{W}=\nabla_{W}\mathcal{L}+2CGG^{T}+\lambda\frac{W}{|W|}$$

Estimated $\lambda_{max}$:

$$\lambda_{max}=\left\{ \begin{aligned} \begin{matrix} \max_{j,k} \frac{{X_{,j}^{(k)}}^{T}Y^{(k)}}{n_{k}} & for least square loss \end{matrix} \\ \begin{matrix} \max_{j, k} \frac{{X_{,j}^{(k)}}^{T}Y^{(k)}}{2n_{k}} & for logit loss \end{matrix} \end{aligned} \right.$$

Where $\sigma_{1}(A)$is the largest singular value of matrix A

Proximal point estimation:

$$\underset{f=\frac{\lambda\left| x \right|}{L}}{\mathrm{prox}} \left( W \right)=sign(W)max\left\{ |W|-\lambda,0 \right\}$$

The method aimed to incorporate the relationships between cohorts as a graph into the model training procedure. ${|\left| GW \right||}_{2}^{2}$ was used for this aim, where G was a pre-defined matrix describing the network structure. More details of G for variant applications can be found in ^8^. ${|\left| W \right||}_{1}$ was used to remove redundant coefficients.$\lambda$ was identified by cross-validation.

## Distributed Optimization Framework

To solve these composite objective functions efficiently in the same framework, we rewrite the objective (1) as

$\min_{x} F\left( x \right)+\lambda\Omega(x)$ (9)

where $F\left( x \right)\mathcal{=L}\left( \theta\right)+CS\left( \theta\right)$ was smooth component function and $\Omega\left( x \right)=\aleph\left( \theta\right)$ was non-smooth

**Solving sub-problem in each iteration**

Given the Lipschitz constant $L$ of the objective function above, the sequence of estimation points $\left\{ x_{0}, x_{1},x_{2},\ldots\right\}$ were found by solving the below iteration-wise sub-problem (9)

$x_{i+1}=\underset{y}{arg min} \mathcal{M}_{L,x_{i}}\left( y \right)$ (10)

$\mathcal{M}_{L, x_{i}}\left( y \right)=F\left( x_{i} \right)+\left\langle\nabla F\left( x_{i} \right), y-x_{i} \right\rangle+\frac{L}{2}\left| \left| y-x_{i} \right| \right|_{2}^{2}+\lambda\Omega\left( x \right)$ (11)

The first three terms on the right side were the second-order approximation of $F\left( . \right)$ using Taylor expansion on point $x_{i}$. After re-organization, we have (12) equal to (10).

$x_{i+1}=\underset{y}{arg min} \frac{L}{2}\left( y-\left( x_{i}-\frac{\nabla F\left( x_{i} \right)}{L} \right) \right)^{2}+\lambda\Omega(x)$ (12)

Since $x_{i}-\frac{\nabla F\left( x_{i} \right)}{L}$ was known after the $i$th iterations, the above problem was applicable to the proximal algorithm framework^10^. For all sparse regularizations ($\Omega(x)$) used in dsMTL, they can be simplified and solved analytically in (13), and the results were derived and summarized above (see the “Proximal point estimation”) for each dsMTL method.

$x_{i+1}=\underset{f=\frac{\lambda\Omega(x)}{L}}{\mathrm{prox}} \left( x_{i}-\frac{\nabla F\left( x_{i} \right)}{L} \right)$ (13)

**Line search**

Since $L$ was unknown in our framework, we estimated it using a backtracking line search approach. Set an increasing sequence of $L\in\left\{ L_{0}, 2L_{0}, 4L_{0}, 16L_{0},\ldots\right\}$given a constant $L_{0}$, the smallest $L$ satisfying the condition $\mathcal{M}_{L, x_{i}}\left( x_{i+1} \right)\geq F\left( x_{i+1} \right)$ was selected. Here, $x_{i+1}$ was determined based on (12).

**Federated computation**

### For supervised MTL, the variable matrix $\boldsymbol{W=p\times t=}\left[ \boldsymbol{w}_{\boldsymbol{,1}}\boldsymbol{,}\boldsymbol{w}_{\boldsymbol{,2}}\boldsymbol{,\ldots,}\boldsymbol{w}_{\boldsymbol{,t}} \right]$, where each column represents one task. So distributed proximal operator was:

$W_{i+1}=\underset{f=\frac{\lambda\Omega(x)}{L}}{\mathrm{prox}} \left( W_{i}-\frac{\nabla F\left( W_{i} \right)}{L} \right)=\underset{f=\frac{\lambda\Omega(x)}{L}}{dist prox} \left( \left[ \begin{matrix} {w_{,1}}_{i}-\frac{\nabla F\left( {w_{,1}}_{i};D^{(1)} \right)}{L}, & \ldots, & {w_{,t}}_{i}-\frac{\nabla F\left( {w_{,t}}_{i};D^{(t)} \right)}{L} \end{matrix} \right] \right)$ (14)

where ${w_{,k}}_{i}-\frac{\nabla F\left( {w_{,k}}_{i} \right)}{L}$ was calculated on server k and sent back. $\left\{ D_{1, \ldots}, D_{t} \right\}$ represented the data on t servers. Similarly, objective function $O\left( W \right)$ has to be evaluated in a distributed fashion,

$$O\left( W \right)=\mathrm{dist} O\left( W \right)=\sum_{k=1}^{t} F\left( W_{,k};D^{(k)} \right)+\lambda\Omega\left( W \right)$$

### For supervised ML, the information aggregation was different. The variable vector $\boldsymbol{w=p\times1}$. The distributed proximal operator is

$$w_{i+1}=\underset{f=\frac{\lambda\Omega(x)}{L}}{\mathrm{prox}} \left( w_{i}-\frac{\nabla F\left( w_{i} \right)}{L} \right)=\underset{f=\frac{\lambda\Omega(x)}{L}}{dist prox} \left( w_{i}-\frac{1}{L}\left( \sum_{j=1}^{t} \nabla\mathcal{L}\left( w_{i};D^{(j)} \right)\frac{n_{j}}{n}+C\nabla\aleph\left( w_{i} \right) \right) \right)$$

where $\nabla\mathcal{L}\left( w_{i};D^{(j)} \right)$ was calculated on server j and sent back. Similarly, objective function $O\left( w \right)$ has to be evaluated in distributed federated fashion,

$O\left( w \right)=\mathrm{dist} O\left( w \right)=\sum_{j=1}^{t} \mathcal{L}\left( w_{i};D^{(j)} \right)\frac{n_{j}}{n}+C\aleph\left( w_{i} \right)+\lambda\Omega\left( w \right)$.

### Accelerated algorithms

**Distributed solver**

**Algorithm 3** Distributed solver of supervised learning methods in dsMTL

**Input**: $\lambda>0$, $L_{0}>0$, $W_{0}$, $maxIter>0$

**Output**:$W_{i+1}$

1: Initialize $W_{1}=W_{0}$, $\alpha_{-1}=\alpha_{0}=0$, and $L=L_{0}$

2: **for** $i=1$ to $maxIter$ **do**

3: Set $S_{i}=W_{i}+\frac{\alpha_{i-1}-1}{\alpha_{i}}\left( W_{i}-W_{i-1} \right)$

4: Find smallest $L\in\left\{ L_{i-1}, 2L_{i-1}, 4L_{i-1}, 16L_{i-1},\ldots\right\}$ such that

$\mathcal{M}_{L, x_{i}}\left( W_{i+1} \right)\geq\mathrm{dist} O\left( W_{i+1} \right)$,

where $W_{i+1}=\mathrm{dist}\underset{f=\frac{\lambda\Omega(x)}{L}}{\mathrm{prox}} \left( W_{i}-\frac{\nabla F\left( W_{i} \right)}{L} \right)$

5: Set $L_{i}=L$, and $\alpha_{i+1}=\frac{1+\sqrt{1+4\alpha_{i}^{2}}}{2}$

6: If termination rule satisfied, **return**

7: **end for**

To accelerate the optimization procedure, we applied Nesterov’s acceleration approach^9,11,12^. In the beginning of iteration$i$, the search point was first defined as the weighted combination of the results from the previous two steps: $S_{i}=\frac{\alpha_{i-1}}{\alpha_{i}}x_{i}+\frac{1-\alpha_{i-1}}{\alpha_{i}}x_{i-1}$. Then the formulas (13) was applied on the $S_{i}$.

**Training for sparse model**

In the high-dimensional data analysis, the performance of sparse ML models was highly related to the accuracy of sparse structure identification, thus $\lambda$ selection was crucial. In dsMTL, we trained the entire regularization tree for a given hyper-paratemer C. Similar to the study^13^, we estimated the $\lambda_{max}$ as the largest $\lambda$ of the sequence from the data. $\lambda_{max}$ was selected by looking for the smallest $\lambda$ such that the equation $\partial_{W}\left( F\left( x \right)+\lambda\Omega\left( x \right) \right)\boldsymbol{\ni0}$ hold. Due to the differential objective functions, $\lambda_{max}$ of classification model was different from that of regression model. The $\lambda_{min}$ was determined based on $\lambda_{max}$, i.e. $0.1\lambda_{max}$. Then the entire sequence was interpolated based on the log scale of $\lambda_{max}$ and $\lambda_{min}$. For each method, $\lambda_{max}$ was theoretically different, and summarized above.

**Algorithm 4** Training procedure of sparse models in dsMTL

**Input**: $\lambda_{1}>\lambda_{2}>\ldots>0$

**Output**:$W_{1}$, $W_{2}$,…

1: Initialize $W_{0}=p\times t=0$

2: **for** $i=\left\{ 1, 2, \ldots\right\}$ **do**

3: $W_{i}=$**Algorithm 3** $\boldsymbol{(}\lambda=\lambda_{i}$,$L_{0}=1$, $W_{0}=W_{i-1}$, $maxIter=100\boldsymbol{)}$

4: **end for**

**Cross-validation**

We set up cross-cohort and in-cohort CV in dsMTL for all ML/MTL methods. For cross-cohort CV, t folds CV were established for *t* cohorts. In fold *i*, cohort *i* was the test cohort and the model was trained on the remaining cohorts. The prediction performances were averaged and used to select $\lambda$. Such CV aimed to identify a $\lambda$ with an optimized generalization performance. For k-folds in-cohort CV, the samples of each cohort were randomly separated into k folds, such that the test folds across cohorts were combined for testing, and the training folds were combined for training. Such CV aimed to identify a $\lambda$ with the most representative sparse model across all cohorts.

# Introduction of DataSHIELD

DataSHIELD^14^ is a platform software supporting federated data analysis without disclosing personally identifiable information. Two modules were included, the Ranalytic environment and the data warehouse opal.

DataSHIELD is efficient for importing/exporting large datasets, e.g., GWAS data requires tens of gigabytes. DataSHIELD was designed to support a wide variety of data types. For this, an architecture package resourcer^15^ developed by the DataSHIELD community was incorporated to facilitate the efficient import and export of large-scale datasets in compressed formats. For example, in DataSHIELD, GWAS data in the PLINK file formats can be read and processed using the software PLINK^16^ as the backend^15^.

To mitigate the risk of sensitive data disclosure, the design of DataSHIELD considers two aspects: software architecture and statistics. The architecture of DataSHIELD provides several non-disclosure mechanisms to improve the system security, such as , 1) DataSHIELD requires the data analysis to only occur behind the firewall; 2) each server is only allowed to communicate with a set of clients with fixed IP addresses; 3) an SSL protocol protects the network communication; 4) an R parser^14^ implemented on the server rejects the calling of unwanted functions; and 5) the so-called ‘disclosure control’^14^ on the server ensures that the returned response does not contain any disclosive information. In addition, several permissions can be set by the data providers to fully control the usage of their data. These permissions describe the degree of accessibility of data and functions on the server i.e., “which users can perform what actions on what data”. In an extremely secure example, a user could be granted to check the summary of a given dataset but cannot perform any actions because no functions were granted. With these settings, DataSHIELD allows customizing the security protection strategies according to the specific requirements of the applications. From a statistics perspective, DataSHIELD assumes that summary statistics are safe to share. Such assumption is quite common in the biomedical field, and there is a large number of websites providing summary data for free downloads, such as the GWAS summary data of certain traits17 and eQTLs of tissues18. Another study19 confirmed the non-disclosure property of DataSHIELD for regression analysis from a biostatistical perspective.

# Data key mechanism

This mechanism as shown in **Supplementary Figure 3** allows the authorized users to obtain the complete model identified by multi-task learning from the server. The administrator generates two keys, stores the local key in the key database, and gives the remote key to a trustworthy user. Then by sending the remote key, the client is seen as the data provider of the server, and can retrieve the complete model from the multi-task learning method.

This mechanism was built for two reasons. 1) The custom-defined functions in DataSHIELD cannot obtain identity information from the users, and 2) specifying the identity of users via the IP address is not sufficiently safe.

**Generation of RNA-seq count data for case study 2**

The RNA-seq count data was generated using the Negative Binomial distribution (NB distribution), which was the most common distribution used to model RNA-seq data. In case study 2, a two-cohort scenario was simulated. Four tests were conducted for different severity of heterogeneity. Here the degree of heterogeneity was characterized by the proportion (20%, 40%, 60% and 80%) of genes in the shared signature over all diagnosis-associated genes: a low proportion represented a high degree of heterogeneity. The simulation procedure contained the following steps. First, the background data of the two cohorts $X_{1}$and $X_{2}$were generated as sampled from the NB distribution ${X_{1}\sim NB}_{p\times n_{1}}(r=2, prob=0.3)$and ${X_{2}\sim NB}_{p\times n_{2}}(r=2, prob=0.3)$, where $p$ was shared gene dimension, $n_{1}$and $n_{2}$ referred to the respective sample size. Then in cohort $i$, the first 50% of samples, $X_{i}[,1:\frac{n_{i}}{2}]$, were selected as patients while the first 50% genes, $X_{i}[1:\frac{p}{2},]$, were selected as the diagnosis-related genes. According to the specific proportion $\varphi\epsilon\left\{ 20\%, 40\%, 60\%, 80\% \right\}$ of shared genes over all signature genes, the shared and cohort-specific disease effect was added to the background data of patients. Specifically, the diagnosis-related effect shared by both cohorts was added as $X_{i}\left[ 1:\frac{p\varphi}{2},1:\frac{n_{i}}{2} \right]=X_{i}\left[ 1:\frac{p\varphi}{2},1:\frac{n_{i}}{2} \right]+\mathrm{NB}_{\frac{p\varphi}{2}\times\frac{n_{i}}{2}}(r=2, p=0.002)$ for cohort $i$. The diagnosis-related effect specific to cohort 1 was added as $X_{1}\left[ \frac{2+p\varphi}{2}:\frac{p(1+\varphi)}{4},1:\frac{n_{1}}{2} \right]=X_{1}\left[ \frac{2+p\varphi}{2}:\frac{p(1+\varphi)}{4},1:\frac{n_{1}}{2} \right]+\mathrm{NB}_{\frac{p(1-\varphi)}{4}\times\frac{n_{1}}{2}}(r=2, p=0.002)$. The diagnosis-related effect specific to cohort 2 was added as $X_{2}\left[ \frac{4+p(1+\varphi)}{4}:\frac{p}{2},1:\frac{n_{2}}{2} \right]=X_{1}\left[ \frac{4+p(1+\varphi)}{4}:\frac{p}{2},1:\frac{n_{2}}{2} \right]+\mathrm{NB}_{\frac{p(1-\varphi)}{4}\times\frac{n_{2}}{2}}(r=2, p=0.002)$. Here the specific effects were not overlapped between cohorts 1 and 2.

# Pre-processing of actual expression datasets

## Microarray data for supervised dsMTL

Four independent cortical microarray gene expression datasets and one blood expression dataset from schizophrenia case-control cohorts were used in this study. Three brain expression datasets were downloaded from the GEO repository with id: GSE53987, GSE21138 and GSE35977. A detailed data description can be found on GEO and the respective original studies^20-22^. The fourth dataset was the HBCC microarray dataset (dbGAP ID: phs000979.v3.p2). The data description and sample acquisition methods can be found on dbGAP and the original publication^23^. The blood sample contained genome-wide gene expression data from lymphoblastoid cell lines of individuals with schizophrenia and controls^24^, and was used to validate the cortical expression signatures.

For GSE53987 and GSE21138, the expression levels were measured using the Affymetrix GeneChip Human Genome U133 Plus 2.0 Array, while the data of GSE35977 was measured using Affymetrix Human Gene 1.0 ST Array. A consistent pre-processing procedure was applied to all datasets. First, the raw data was extracted by the function *ReadAffy*() of the R package affy 1.64.0^25^, followed by the rma^26^ (Robust Multi-array Average) procedure for normalization. Values from multiple probes related to the same gene were averaged. Second, subjects with ages < 18 were excluded. Third, outliers were deleted as those outside of four standard deviations from the mean of the first two principal components. Fourth, 10 surrogate variables were determined using SVA^27^ from the R package sva 3.34.0. Fifth, multiple linear regression analysis was used to correct for the effect of potential confounders with the covariates age, age^2^, sex, PMI, pH, RIN, batch ID and 5 surrogate variables. Sixth, the resulting expression genes were z-standardized.

HBCC data was normalized and quality controlled as previously described^23^. First, we extracted the raw dlpfc expression data using the function *read.idat*() from the R package limma 3.42.2 ^28^. Second, we corrected for background noise using the negative probes followed by quantile normalization and log-transformation. Third, we retained the robustly expressed probes as those with a detection p-value<0.01 in at least half of the individuals. Prior to sva analysis, the missing “pH” and “PMI” values were imputed using the average of available data. The covariates contained age, age^2^, sex, PMI, pH, RIN, ethnicity and 5 surrogate variables. The cohort contained 321 healthy controls and 191 patients with schizophrenia. All four datasets shared 8013 overlapping genes.

The blood expression dataset was pre-processed as previously suggested^24^. For each batch, the robustly-expressed probes (detection False Discovery Rate (FDR) <0.05) were retained for analysis. For each batch, the expression matrix was shifted such that the lowest expression value was 1, log2-transformed and quantile normalized. The two batches were subsequently combined. Too young (age<18) and old (age>65) subjects were removed from the data. Outliers were removed as those outside of four deviations from the mean of the first two principal components. Five hidden variables were determined using SVA^27^. Downstream statistical analysis of this cohort included age, age2, sex, viral load, energy level, growth rate, transformation site, background intensity, batch ID and five hidden variables as covariates. In the end, 413 schizophrenia patients and 446 healthy controls were included in the analysis.

## RNA-seq data for unsupervised dsMTL

Two processed RNA-seq case-control cohorts comprising patients with schizophrenia (GSE164376^29^ ) and bipolar disorder (GSE134497^30^) were retrieved from the GEO database and converted into a matrix format for the analysis.

# Simulation data Analysis

**Two case studies**

**Case study 1.** In this case study, the heterogeneous cohorts were generated with the same set of outcome-associated genes. These, however, showed different directionality of their respective associations with the outcome. A three-server scenario was simulated. 150 out of 500 features with random signs across cohorts were simulated. Seven tests were created for simulating different n/p ($\frac{sample size}{gene number}$) ratios. The n/p ratio was $\left\{ 1.2, 1, 0.9, 0.6, 0.5, 0.3, 0.15 \right\}$ with the number of subjects $\left\{ 600, 500, 450, 300, 250, 150, 75 \right\}$ for each test. 500 genes were created for each server. The test sample consisted of 200 subjects for each server. Data were generated as follows:

Given gene number$p=500$, the models of three cohorts were $\left\{ w^{(1)}, w^{(3)},w^{(3)} \right\}$ where $w^{(.)}=p\times1$. A shared signature comprising 150 genes was generated for each $w^{(.)}$ but with random signs, ${w^{(.)}}_{i}=\left\{ \begin{matrix} 2\times(\rho-0.5)\times N(1, 0.1) & 1<i<150 \\ 0 & \mathrm{others} \end{matrix} \right.$, $\rho\sim\mathrm{Bernoulli}(\frac{1}{2})$. The expression values of each subject across cohorts were generated as $x=1\times p$ $\mathrm{where}x_{j}\sim N(0,1)$. The numeric outcome (e.g. symptom severity) $y=xw^{(i)}$ in cohort $i$ was standardized in a normal distribution $N\left( 0, 1 \right)$, then model-irrelevant noise with 50% of the variance of the true signal was added $y=y+N\left( 0, 0.5 \right)$.

dsMTL_L21 and dsLasso were trained as the federated learning system, and the hyper-parameter was selected using 10 fold in-cohort cross-validation. For glmnet, the ensemble technique was only applied on the gene selection due to the consistent gene set of their signatures. The mean squared error (mse) was used to measure prediction performance. To account for the sampling variance, we repeated each analysis 100 times.

**Case study 2.** In this case study, two heterogeneous RNA-seq cohorts were created to simulate a comorbidity analysis, where the genes were separated to be part of either a shared signature among cohorts, cohort-specific signatures or diagnosis-unassociated genes. The dsMTL_iNMF was compared to the ensemble of local NMF regarding the selection accuracy of shared/cohort-specific genes, in particular impacted by the severity of heterogeneity. Here the severity of heterogeneity refers to the proportion of the genes harbored by the shared signature over all diagnosis-associated genes. The data simulation protocol for RNA-seq data can be found in the above sections.

A two-server scenario was simulated. As shown in **Supplementary Table 2**, for the data of each server, 1000 genes and 200 subjects were simulated, 50% of the genes were diagnosis-unassociated and the remaining genes were part of the disease signature. The genes comprised by shared signatures were identical for data of two servers, and the genes comprised by cohort-specific signatures did not overlap. The case-control ratio was balanced for each server. Four tests were performed by varying the proportion of genes in the shared signature over all diagnosis-associated genes from 20% to 80%.

The training of dsMTL_iNMF results in three outputs related to the original input data: the shared gene ‘exposure’ (H), cohort-specific gene ‘exposure’ (V) and sample ‘exposure’ (W). We measured the association between the sample exposure and the diagnosis as the weight of each latent factor. The shared (or specific) gene signature was identified as the weighted summation of the shared (or specific) gene exposures over latent factors. To quantify the important genes related to a given signature, we binarized the gene signature according to the mean (0-1 vector, the values were larger than the mean were assigned to the identified genes). To assess the performance of the gene identification, we associated the selected genes with the ground truth (0-1 vector, signature genes were 1). The assessment was applied to shared and cohort-specific genes in parallel. Based on this metric, three gene sets were derived as output from dsMTL_iNMF, called dsMTL_iNMF-H, dsMTL_iNMF-V1 and dsMTL_iNMF-V2, and these related to the shared, cohort 1 specific and cohort 2 specific gene signature, respectively. The same strategy was applied to analyze the ensemble of local NMF models. For each cohort, the specific gene signature was the weighted summation of gene exposure over latent factors, and then binarized as the specific gene set (called local-NMF1 and local-NMF2). The shared gene signature was identified as the sum of the specific gene signature over cohorts, and then binarized as the shared gene set (NMF-bagging). We then compared 1) NMF-bagging and dsMTL_iNMF-H for the accuracy related to the isolation of shared genes; 2) dsMTL_iNMF-V1 and local-NMF1 as well as dsMTL_iNMF-V2 and local-NMF2 for the accuracy of isolating cohort-specific genes.

**Scalability analysis up to 20 servers**

We used DSLite^15^ to simulate DataSHIELD servers in one machine. For $k\in\left\{ 1,2,\ldots,20 \right\}$ servers, we create k datasets with a fixed dimensionality (p=100) and varied subject number ($n\in\left\{ 20,50,70,120 \right\}$). The algorithms dsMTL_L21 and dsLasso were trained on these datasets with the same setting to guarantee comparable results. For example, the same configurations are starting point (0 vector), stopping criteria (ter=2), tolerance (tol=0.001) and maximized the number of iterations (50), hyper-parameter (C=0.1), the number of lambdas (50) and the ratio of lambda (0.001). Note, the dataset simulated for each algorithm followed the assumption of the algorithm.

# Actual data analysis

**Computational speed analysis**

**Supervised dsMTL.** We aimed at identifying the efficiency of supervised dsMTL using real molecular data and given the real network latency. Four independent schizophrenia case-control cohorts were used for this analysis. The training cohorts consisted of three datasets comprising prefrontal cortex gene expression data (available from the GEO repository under accession numbers GSE53987, GSE21138 and GSE35977). The dataset used for algorithm testing was from the HBCC (n=422) cohort comprising genome-wide gene expression data quantified by microarray (dbGAP ID: phs000979.v3.p2). As shown in **Supplementary Table 3**, three servers were used for training algorithms. Two servers were held at the Central Institute of Mental Health, Mannheim while the third was positioned at the BioQuant institute, Heidelberg.

Using these datasets, we repeated a previously described analysis^31^, in order to evaluate computational speed in a federated analysis setting. Here we show the formulation of the mean regularized MTL using dsMTL_net:

The cohort-level batch effect was assumed to be Gaussian noise affecting the true coefficient of gene i and cohort j $w_{ij}=w_{i}+\epsilon_{j}$, $\epsilon_{j}\in N(\mu,\sigma)$. Hence, the average model $\bar{w_{i}}$ across cohorts was an unbiased estimator for the true coefficient, and therefore the squared penalty $\left| w_{ij}-\bar{w_{i}} \right|^{2}$ was incorporated to penalize the departure of each model j to the mean. The complete formulation was

$\min_{W} \sum_{k=1}^{3} \sum_{i=1}^{n_{k}} \frac{1}{n_{k}}\log(1+e^{-Y_{i}^{(k)}\left( X_{i}^{(k)}W_{,k} \right)})+\lambda{|\left| W \right||}_{1}+C{|\left| WG \right||}_{2}^{2}$,

where $G=\left[ \begin{matrix} \begin{matrix} \frac{2}{3} & 0 & \frac{-1}{3} \\ \frac{-1}{3} & \frac{2}{3} & 0 \\ 0 & \frac{-1}{3} & \frac{2}{3} \end{matrix} & \begin{matrix} \frac{2}{3} & \frac{-1}{3} & 0 \\ 0 & \frac{2}{3} & \frac{-1}{3} \\ \frac{-1}{3} & 0 & \frac{2}{3} \end{matrix} \end{matrix} \right]$

**Unsupervised dsMTL.** Here, we analyzed the time efficiency in applying dsMTL_iNMF on two real datasets based on the real network latency. This analysis aimed to identify the shared gene expression signatures between schizophrenia (GSE164376^29^ ) and bipolar disorder (GSE134497^30^). As shown in **Supplementary Table 4**, the data were stored on servers in Mannheim and Heidelberg. We applied dsMTL_iNMF to train the model based on the two datasets and record the time consumption.

**Cross-tissue prediction and pathway enrichment analysis**

To test the cross-tissue generalizability and to explore the biological plausibility of the identified dsMTL_Net model, we predicted the model in data from blood samples and performed pathway-related enrichment analysis. First, a new dsMTL_Net model was trained based on the brain expression datasets GSE53987, GSE21138 and GSE35977 with a more conservative lambda to remove a larger number of genes. Then the model was predicted in the blood expression sample (n=859) to obtain the prediction scores. Finally, an association test between the prediction scores and diagnosis was performed to show cross-tissue predictability.

The top-ranked 200 genes were selected as the gene signatures for the enrichment analysis. The *enrichGO* function from clusterProfiler^32^ package was applied to test the enrichment of the selected genes in different GO pathways. The pathways with too few (i.e., less than 10) or too many (i.e., more than 500) genes were excluded from the analysis.

For the unsupervised method, the pathway analysis was applied to interpret both the shared as well as disease specific genes. The resulting genes were sent to pathway analysis.

**Reproducibility analysis of signatures identification**

A recent review^33^ showed that the expression studies of schizophrenia performed over the past 15 years have provided little consistent evidence for specific genes to be reproducibly associtated with schizophrenia. This might be due to the data scarcity and the heterogeneities of schizophrenia expression studies. We previously found^31^ that MTL was able to identify the more reproducible schizophrenia signatures compared to conventional machine learning. Here we aimed to show that gene signatures found by federated MTL were more reproducible in independent data than those found by the ML-bagging method.

**Method.** We started with four processed cortex expression datasets: GSE53987, GSE21138, GSE35977 and HBCC. These datasets were categorized into two distinct dataset groups, as shown in table 2. Each group contained two datasets. We trained dsMTL_Net and glmnet models for each group and selected the top-ranked 200 genes as the gene signatures. The ranking criterium was the absolute value of the average coefficients of a given gene across datasets. This training and gene selection procedure was repeated 100 times, in order to account for sampling variability. The results were then averaged across the repetitions. We then compared the selected genes across dataset groups, in order to quantify the number of overlapping gene signatures and genes with “consistent directions”. This refers to the number of overlapping signatures that demonstrated a consistent directionality of the outcome-association.

**Supplementary Figures**


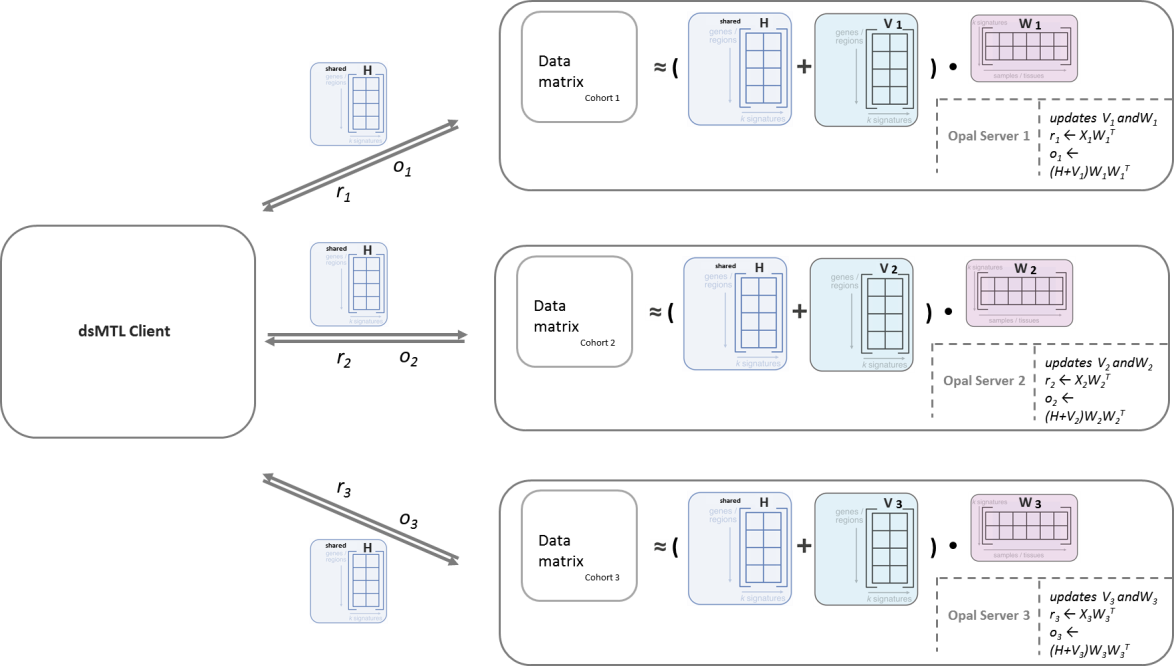


**Supplementary Figure 1.** Communications between the client and opal servers for dsMTL_iNMF


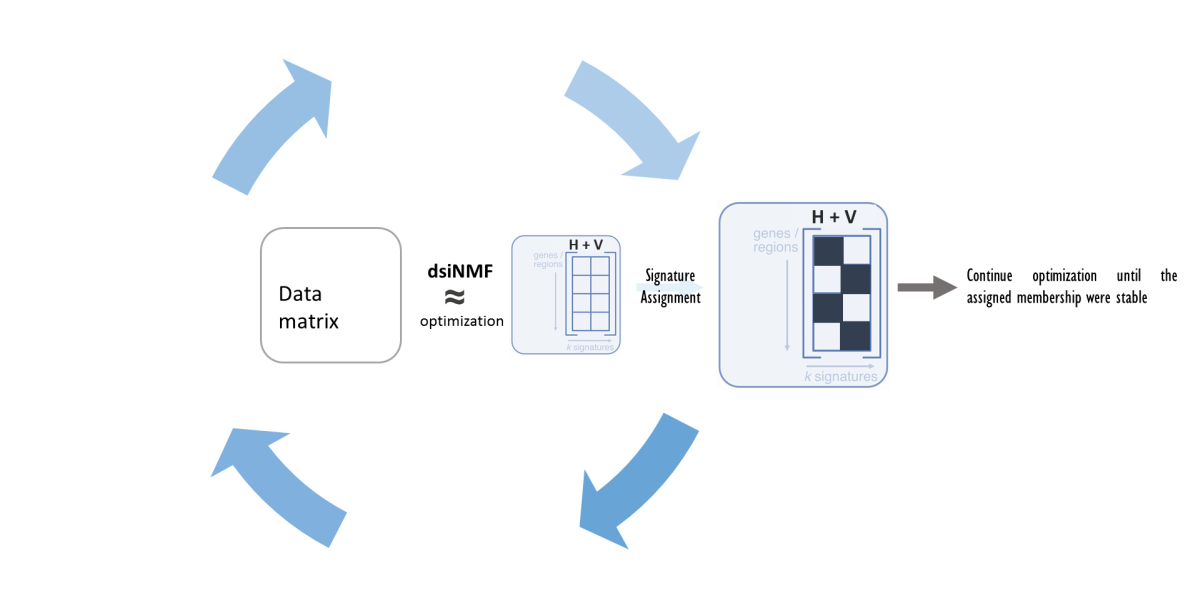


**Supplementary Figure 2**. Schematic illustration of cluster membership optimization.


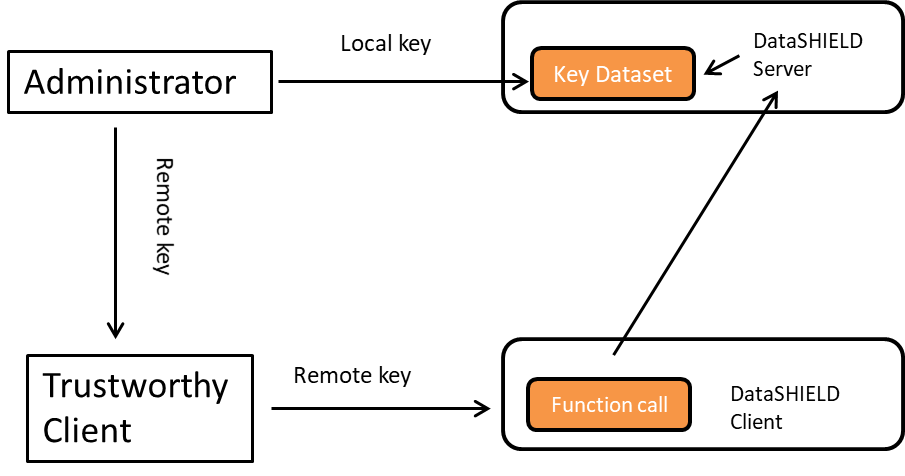


**Supplementary Figure 3**. Schematic illustration of the data key mechanism

**
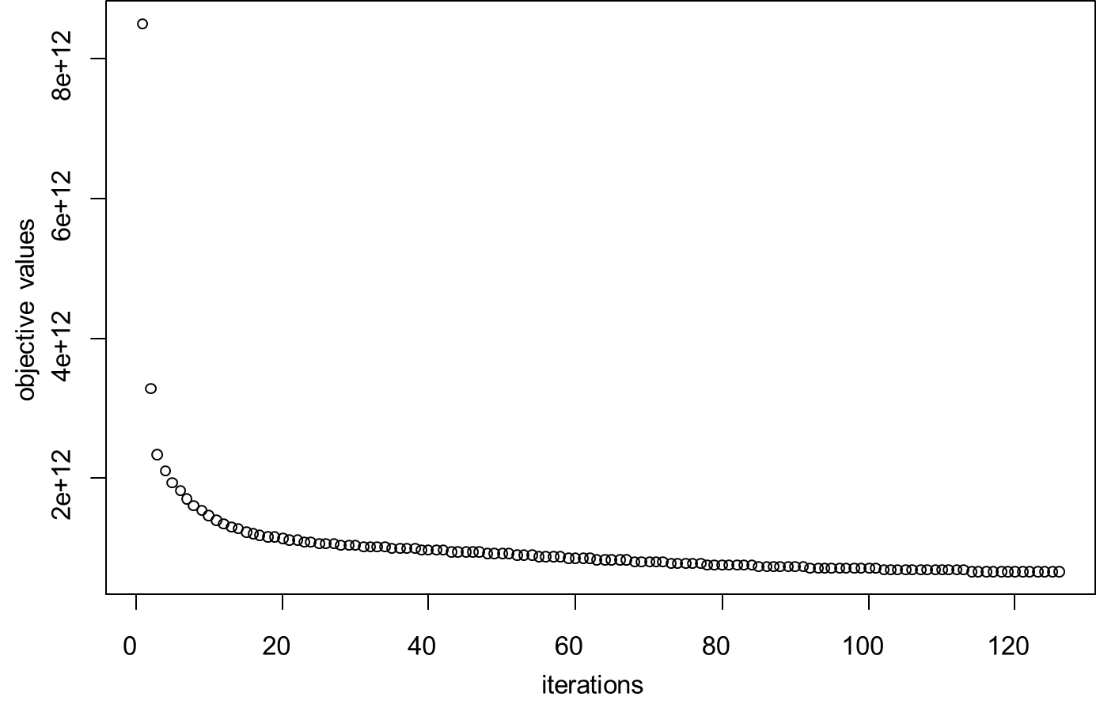
**

**Supplementary Figure 4**: the curve of objectives training dsMTL_iNMF with an initialization in case study 5. 100 iterations were sufficient to converge to a solution with an acceptable precision.


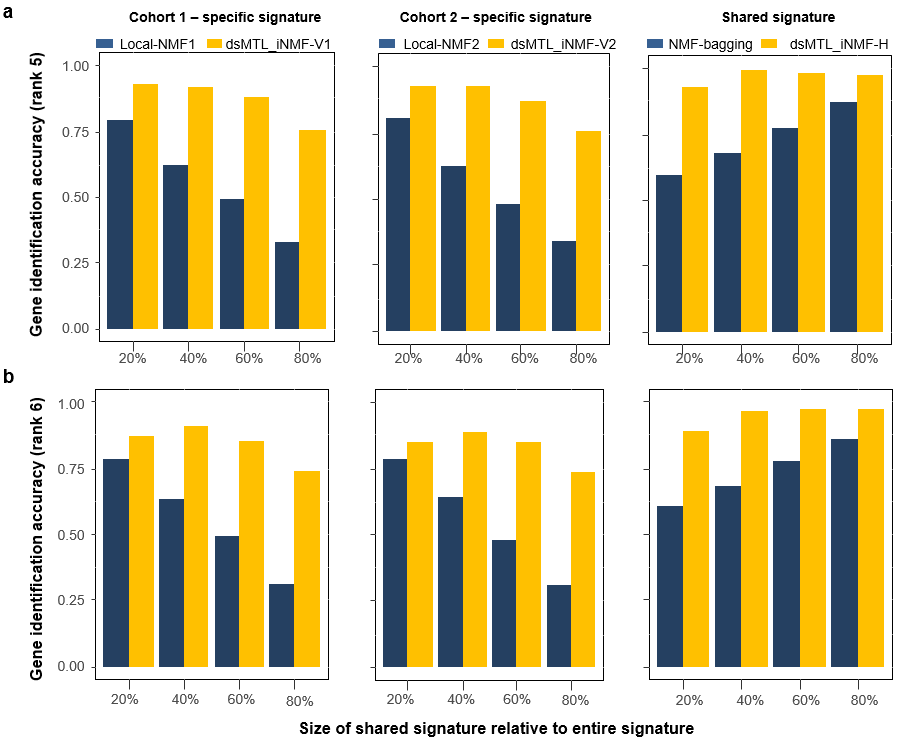


**Supplementary Figure 5. The gene identification accuracy for shared and specific signatures using simulated data using** rank=5 (**a**) and rank=6 (**b**) as model parameters.

**Supplementary Tables**

|  | Unsupervised Learning | Supervised Learning | |
| --- | --- | --- | --- |
|  | Matrix factorization | Regression | Classification |
| Model | $\left[ X_{1}, \ldots,X_{k}, \ldots X_{t} \right]=\left[ (H+{Hv}_{1})\times W_{1}, \ldots, (H+{Hv}_{t})\times W_{t} \right])$ | $f\left( x \right)=xw$ | $P\left( x \right)=\frac{1}{1+e^{-(xw)}}$ |
| Loss function | $\min_{\begin{aligned} H, \\ W_{1}\ldots,W_{t}, \\ V_{1}\ldots,V_{t} \\ \end{aligned}} \sum_{k=1}^{t} {\vert\vert X_{k}-(H+V_{k})W_{k}\vert\vert}_{F}^{2}$ | $\min_{w} \frac{1}{2n}\sum_{i=1}^{n} {\vert\vert y_{i}-x_{i}w\vert\vert}^{2}$ | $\min_{w} \frac{1}{n}\sum_{i=1}^{n} \log(1+e^{-y_{i}\left( x_{i}w \right)})$ |
| Gradient | $\nabla_{H_{i,j}}=2\sum_{k=1}^{t} (H_{i,j}W_{k_{i}}W_{k_{i}}^{T}-X_{k_{i}}W_{k_{i}}^{T})$  $\nabla_{{W_{k}}_{i,j}}=2\sum_{m=1}^{n_{t}} \left( HV_{k} \right)_{m,i}\left[ \left( HV_{k} \right)_{m,i}W_{k_{ij}}-X_{k_{m,j}} \right]$  $\nabla_{{V_{k}}_{i,j}}=2(V_{i,j}W_{k_{i}}W_{k_{i}}^{T}-X_{k_{i}}W_{k_{i}}^{T})$ | $\nabla_{w}=\frac{1}{n}\left( x^{T}xw-x^{T}Y \right)$ | $\nabla_{w}=-\frac{1}{n}X^{T}\times\left[ \begin{matrix} \frac{y_{1}}{1+e^{y_{1}\left( x_{1}w \right)}} \\ \ldots\\ \frac{y_{n}}{1+e^{y_{n}\left( x_{n}w \right)}} \end{matrix} \right]$ |

**Supplementary Table 1.** Summaries of loss functions used in dsMTL

| Index of test | Proportion of homogenous signatures | Number of samples | Number of features | Number of signatures | Proportion of patients |
| --- | --- | --- | --- | --- | --- |
| 1 | 20% | 200 | 1000 | 500 | 50% |
| 2 | 40% |  |  |  |  |
| 3 | 60% |  |  |  |  |
| 4 | 80% |  |  |  |  |

**Supplementary Table 2.** The simulation data of each server. These parameters were same to each of two servers. The only difference is the set of heterogeneous signatures.

|  | | Server 1 | Server 2 | Server 3 | Client |
| --- | --- | --- | --- | --- | --- |
| Type | | Training | Training | Training | Testing |
| Location | | Mannheim | Mannheim | Heidelberg | Mannheim |
| Hardware | CPU | I7-4790 3.6GHz | I7-4790 3.6GHz | Intel Xeon 2.4 GHz | I7-4790 3.6GHz |
|  | Memory | 4G | 4G | 4G | 16G |
| ID | | GSE35977 | GSE21138 | GSE53987 | HBCC |
| Number of Subjects | | 101 | 59 | 34 | 422 |
| Number of Genes | | 8013 | 8013 | 8013 | 8013 |

**Supplementary Table 3.** Client-server architecture for the real data analysis.

|  | | Server 1 | Server 2 |
| --- | --- | --- | --- |
| Type | | Training | Training |
| Location | | Heidelberg | Mannheim |
| Hardware | CPU | Intel Xeon 2.4 GHz | I7-4790 3.6GHz |
|  | Memory | 4G | 4G |
| ID | | GSE164376 | GSE134497 |
| Number of Subjects | | 17 | 16 |
| Number of Genes | | 15215 | 15215 |

**Supplementary Table 4.** Details of server configurations used for real data analysis.

| ONTOLOGY | ID | Description | GeneRatio | BgRatio | pvalue | p.adjust | qvalue | geneID |
| --- | --- | --- | --- | --- | --- | --- | --- | --- |
| BP | GO:0006882 | cellular zinc ion homeostasis | 7/181 | 24/7141 | 1.4386E-06 | 0.00151881 | 0.00138609 | S100A8/MT2A/S100A9/MT1X/MT1E/MT1M/MT3 |
| BP | GO:0055069 | zinc ion homeostasis | 7/181 | 24/7141 | 1.4386E-06 | 0.00151881 | 0.00138609 | S100A8/MT2A/S100A9/MT1X/MT1E/MT1M/MT3 |
| BP | GO:0061687 | detoxification of inorganic compound | 5/181 | 10/7141 | 2.2509E-06 | 0.00151881 | 0.00138609 | MT2A/MT1X/MT1E/MT1M/MT3 |
| BP | GO:0097501 | stress response to metal ion | 5/181 | 10/7141 | 2.2509E-06 | 0.00151881 | 0.00138609 | MT2A/MT1X/MT1E/MT1M/MT3 |
| BP | GO:0071280 | cellular response to copper ion | 5/181 | 15/7141 | 2.4187E-05 | 0.01087994 | 0.00992924 | MT2A/MT1X/MT1E/MT1M/MT3 |
| BP | GO:0071294 | cellular response to zinc ion | 5/181 | 15/7141 | 2.4187E-05 | 0.01087994 | 0.00992924 | MT2A/MT1X/MT1E/MT1M/MT3 |
| BP | GO:0098754 | detoxification | 9/181 | 67/7141 | 4.2637E-05 | 0.01564485 | 0.01427778 | MT2A/S100A9/MT1X/MT1E/NQO1/SESN1/MT1M/MGST1/MT3 |
| BP | GO:0010043 | response to zinc ion | 6/181 | 27/7141 | 4.6372E-05 | 0.01564485 | 0.01427778 | S100A8/MT2A/MT1X/MT1E/MT1M/MT3 |
| BP | GO:0071276 | cellular response to cadmium ion | 5/181 | 18/7141 | 6.4862E-05 | 0.0194514 | 0.01775171 | MT2A/MT1X/MT1E/MT1M/MT3 |
| BP | GO:0040008 | regulation of growth | 21/181 | 332/7141 | 9.1561E-05 | 0.02471234 | 0.02255294 | MTM1/SEMA4B/ISLR2/S100A8/MT2A/DAB2/S100A9/MT1X/MT1E/CDC42/PTK2B/PSRC1/TKT/FGF2/SEMA5A/WFS1/PAFAH1B1/MT1M/BBS2/MT3/ADNP2 |
| BP | GO:0046688 | response to copper ion | 5/181 | 21/7141 | 0.00014481 | 0.03553022 | 0.03242555 | MT2A/MT1X/MT1E/MT1M/MT3 |
| BP | GO:0046916 | cellular transition metal ion homeostasis | 8/181 | 63/7141 | 0.00017171 | 0.03862108 | 0.03524632 | S100A8/MT2A/S100A9/MT1X/MT1E/SCO2/MT1M/MT3 |

**Supplementary Table 5.** The complete list of significant pathways for actual data analysis. The dsMTL_Net model was trained on three cortex expression datasets of schizophrenia. The top-ranked 200 genes were selected as the gene signatures and used for pathway enrichment analysis.

**References**

1. Cao H, Zhou J, Schwarz E. RMTL: An R Library for Multi-Task Learning. *Bioinformatics.* 2018.

2. Quintero A, Hubschmann D, Kurzawa N, et al. ShinyButchR: Interactive NMF-based decomposition workflow of genome-scale datasets. *Biology methods & protocols.* 2020;5(1):bpaa022.

3. Yang Z, Michailidis G. A non-negative matrix factorization method for detecting modules in heterogeneous omics multi-modal data. *Bioinformatics.* 2016;32(1):1-8.

4. Tibshirani R. Regression shrinkage and selection via the lasso: a retrospective. *Journal of the Royal Statistical Society: Series B (Statistical Methodology).* 2011;73(3):273-282.

5. Zou H, Hastie T. Regularization and variable selection via the elastic net. *Journal of the Royal Statistical Society: Series B (Statistical Methodology).* 2005;67(2):301-320.

6. Liu J, Ji S, Ye J. Multi-task feature learning via efficient l2, 1-norm minimization. Paper presented at: Proceedings of the Twenty-Fifth Conference on Uncertainty in Artificial Intelligence2009.

7. Pong TK, Tseng P, Ji S, Ye J. Trace Norm Regularization: Reformulations, Algorithms, and Multi-Task Learning. *SIAM Journal on Optimization.* 2010;20(6):3465-3489.

8. Cao H, Schwarz E. An Tutorial for Regularized Multi-task Learning using the package RMTL. The Comprehensive R Archive Network. Accessed.

9. Beck A, Teboulle M. A fast iterative shrinkage-thresholding algorithm for linear inverse problems. *SIAM journal on imaging sciences.* 2009;2(1):183-202.

10. Parikh N, Boyd S. Proximal algorithms. *Foundations and Trends® in Optimization.* 2014;1(3):127-239.

11. Nesterov Y. Gradient methods for minimizing composite functions. *Mathematical Programming.* 2012;140(1):125-161.

12. Liu J, Jieping Y. Efficient L1/Lq Norm Regularization.

13. Friedman J, Hastie T, Tibshirani R. Regularization Paths for Generalized Linear Models via Coordinate Descent. *Journal of Statistical Software.* 2010;33(1).

14. Wilson RC, Butters OW, Avraam D, et al. DataSHIELD – New Directions and Dimensions. *Data Science Journal.* 2017;16.

15. Marcon Y, Bishop T, Avraam D, et al. Orchestrating privacy-protected big data analyses of data from different resources with R and DataSHIELD. *PLoS computational biology.* 2021;17(3):e1008880.

16. Purcell S, Neale B, Todd-Brown K, et al. PLINK: a tool set for whole-genome association and population-based linkage analyses. *American journal of human genetics.* 2007;81(3):559-575.

17. Zheng J, Erzurumluoglu AM, Elsworth BL, et al. LD Hub: a centralized database and web interface to perform LD score regression that maximizes the potential of summary level GWAS data for SNP heritability and genetic correlation analysis. *Bioinformatics.* 2017;33(2):272-279.

18. Consortium GT. Human genomics. The Genotype-Tissue Expression (GTEx) pilot analysis: multitissue gene regulation in humans. *Science.* 2015;348(6235):648-660.

19. Jones EM, Sheehan NA, Masca N, Wallace SE, Murtagh MJ, Burton PR. DataSHIELD – shared individual-level analysis without sharing the data: a biostatistical perspective. *Norsk Epidemiologi.* 2012;21(2).

20. Lanz TA, Reinhart V, Sheehan MJ, et al. Postmortem transcriptional profiling reveals widespread increase in inflammation in schizophrenia: a comparison of prefrontal cortex, striatum, and hippocampus among matched tetrads of controls with subjects diagnosed with schizophrenia, bipolar or major depressive disorder. *Translational psychiatry.* 2019;9(1):151.

21. Tang B, Capitao C, Dean B, Thomas EA. Differential age- and disease-related effects on the expression of genes related to the arachidonic acid signaling pathway in schizophrenia. *Psychiatry research.* 2012;196(2-3):201-206.

22. Chen C, Cheng L, Grennan K, et al. Two gene co-expression modules differentiate psychotics and controls. *Molecular psychiatry.* 2013;18(12):1308-1314.

23. Fromer M, Roussos P, Sieberts SK, et al. Gene expression elucidates functional impact of polygenic risk for schizophrenia. *Nature neuroscience.* 2016;19(11):1442-1453.

24. Sanders AR, Goring HH, Duan J, et al. Transcriptome study of differential expression in schizophrenia. *Human molecular genetics.* 2013;22(24):5001-5014.

25. Gautier L, Cope L, Bolstad BM, Irizarry RA. affy--analysis of Affymetrix GeneChip data at the probe level. *Bioinformatics.* 2004;20(3):307-315.

26. Bolstad BM, Irizarry RA, Astrand M, Speed TP. A comparison of normalization methods for high density oligonucleotide array data based on variance and bias. *Bioinformatics.* 2003;19(2):185-193.

27. Leek JT, Johnson WE, Parker HS, Jaffe AE, Storey JD. The sva package for removing batch effects and other unwanted variation in high-throughput experiments. *Bioinformatics.* 2012;28(6):882-883.

28. Ritchie ME, Phipson B, Wu D, et al. limma powers differential expression analyses for RNA-sequencing and microarray studies. *Nucleic acids research.* 2015;43(7):e47-e47.

29. A; K, R; K. GSE164376 dataset. <https://www.ncbi.nlm.nih.gov/geo/query/acc.cgi?acc=GSE164376>. Published 2021. Accessed.

30. Kathuria A, Lopez-Lengowski K, Vater M, McPhie D, Cohen BM, Karmacharya R. Transcriptome analysis and functional characterization of cerebral organoids in bipolar disorder. *Genome medicine.* 2020;12(1):34.

31. Cao H, Meyer-Lindenberg A, Schwarz E. Comparative Evaluation of Machine Learning Strategies for Analyzing Big Data in Psychiatry. *International journal of molecular sciences.* 2018;19(11).

32. Yu G, Wang LG, Han Y, He QY. clusterProfiler: an R package for comparing biological themes among gene clusters. *Omics : a journal of integrative biology.* 2012;16(5):284-287.

33. Merikangas AK, Shelly M, Knighton A, Kotler N, Tanenbaum N, Almasy L. What genes are differentially expressed in individuals with schizophrenia? A systematic review. *Molecular psychiatry.* 2022.
