# Supplementary figures and images for: dsMTL: a computational framework for privacy-preserving, distributed multi-task machine learning
Source: Bioinformatics. 2022 Sep 8;38(21):4919–26. doi: 10.1093/bioinformatics/btac616 (PMC9620828; doi:10.1093/bioinformatics/btac616)

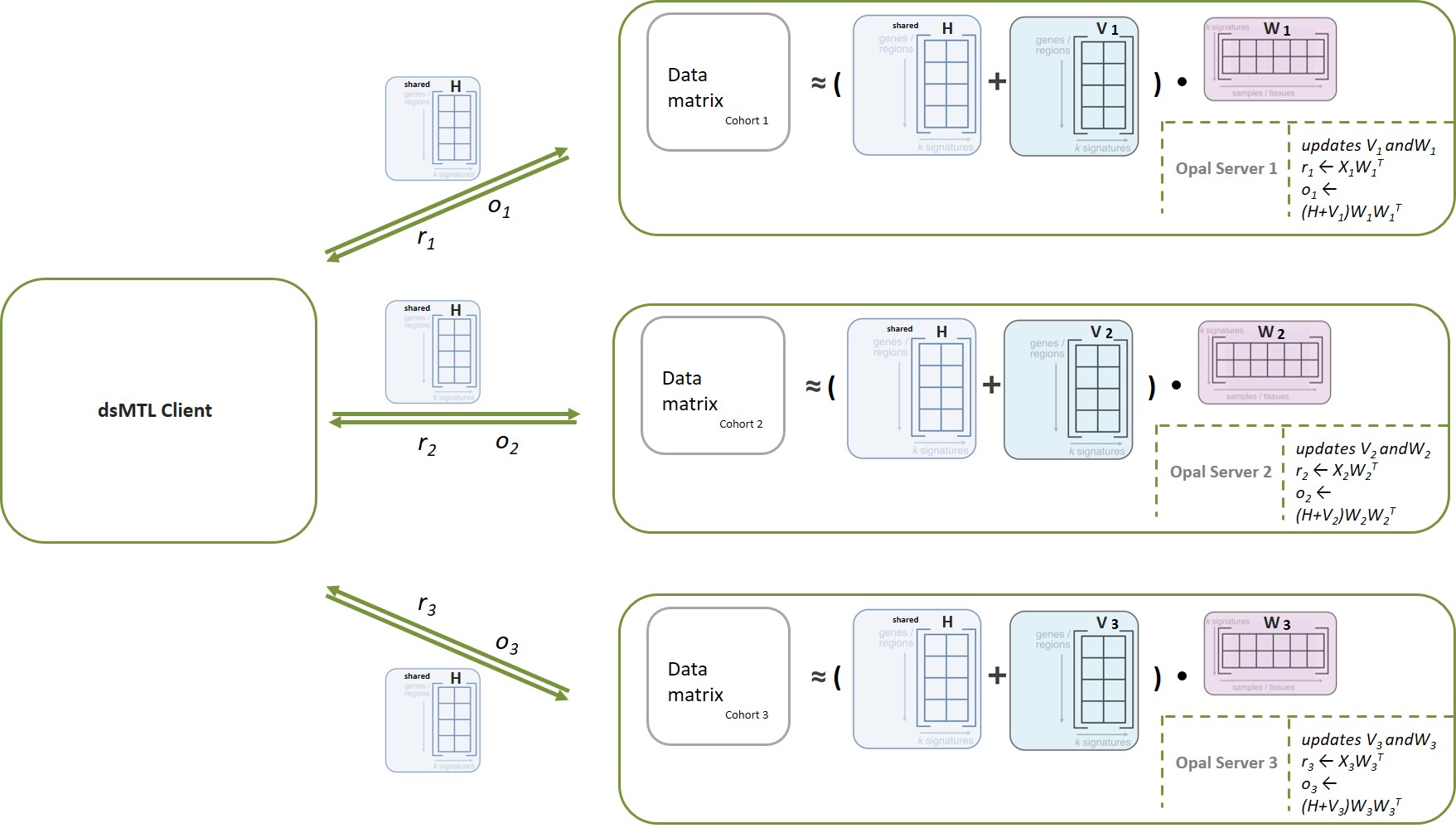

Supplement: btac616_Supplementary_Data [file btac616_supplementary_data.zip › Supplementary Figure 1.jpg]

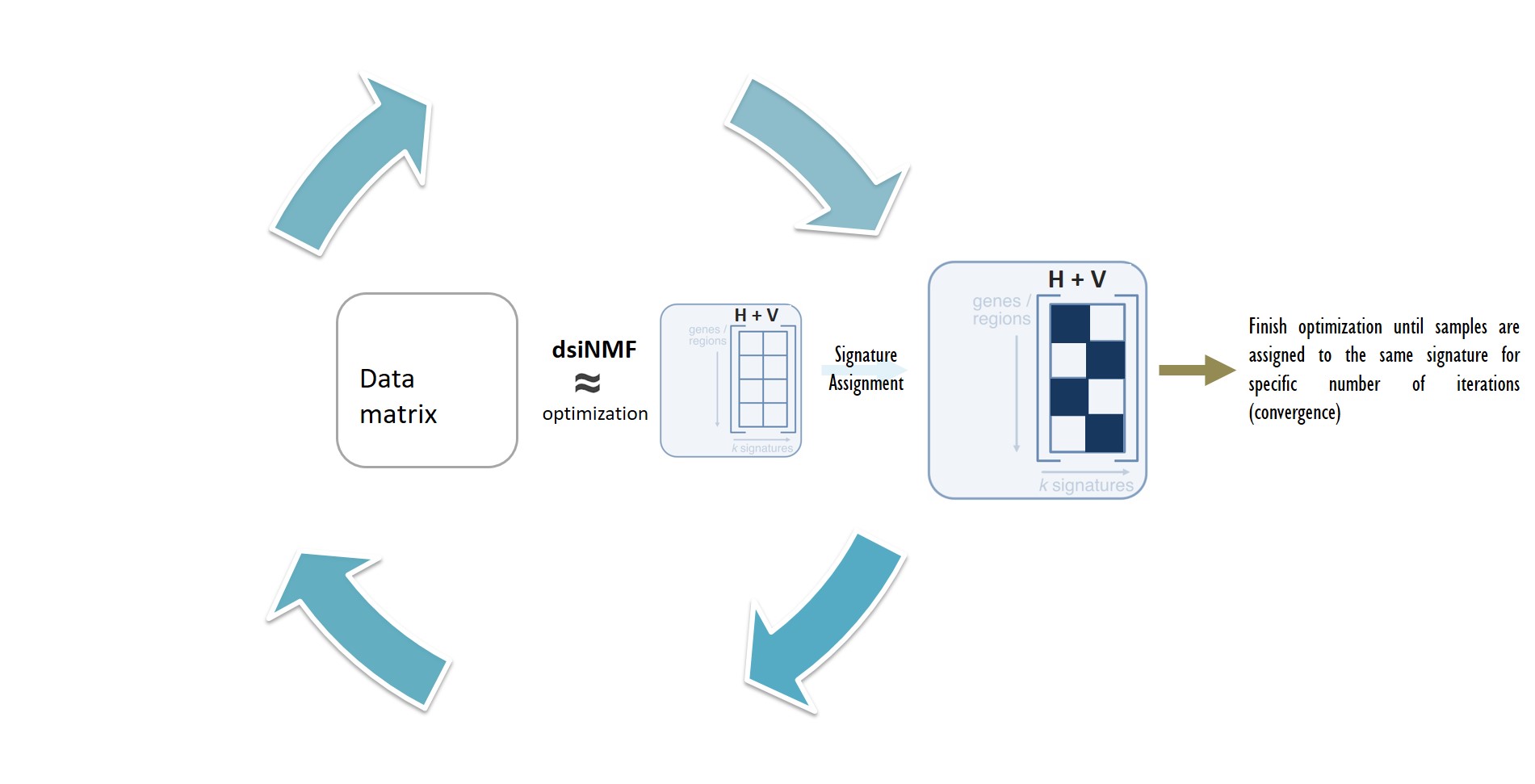

Supplement: btac616_Supplementary_Data [file btac616_supplementary_data.zip › Supplementary Figure 2.jpg]

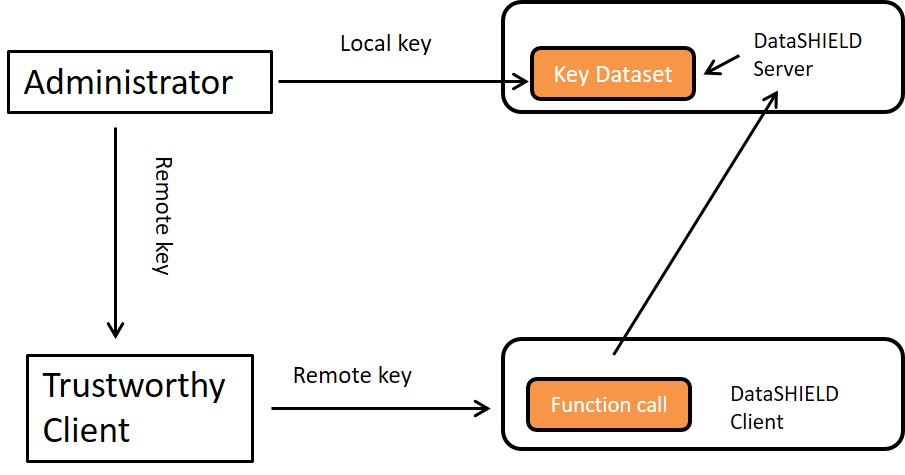

Supplement: btac616_Supplementary_Data [file btac616_supplementary_data.zip › Supplementary Figure 3.jpg]

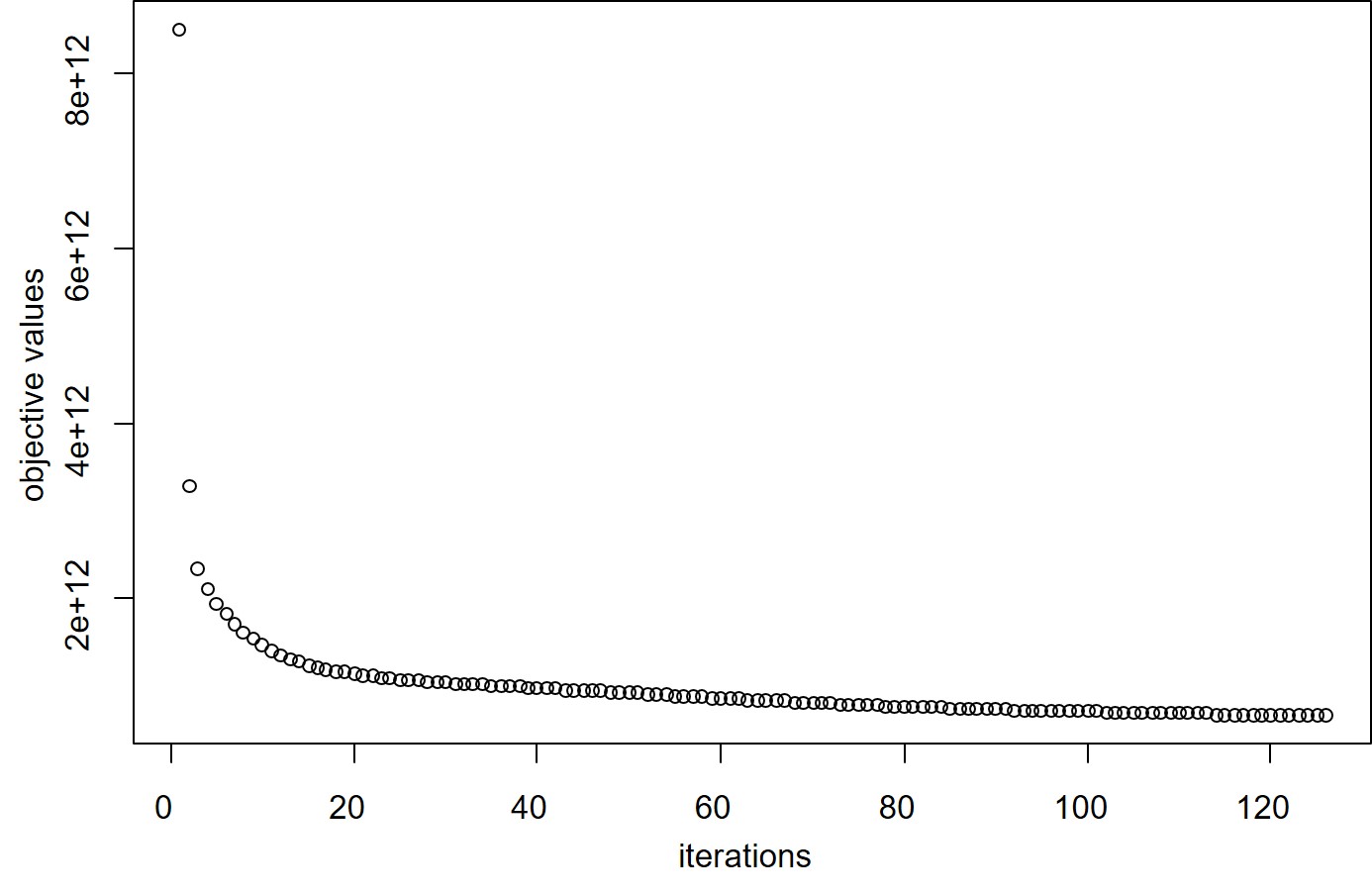

Supplement: btac616_Supplementary_Data [file btac616_supplementary_data.zip › Supplementary Figure 4.jpg]

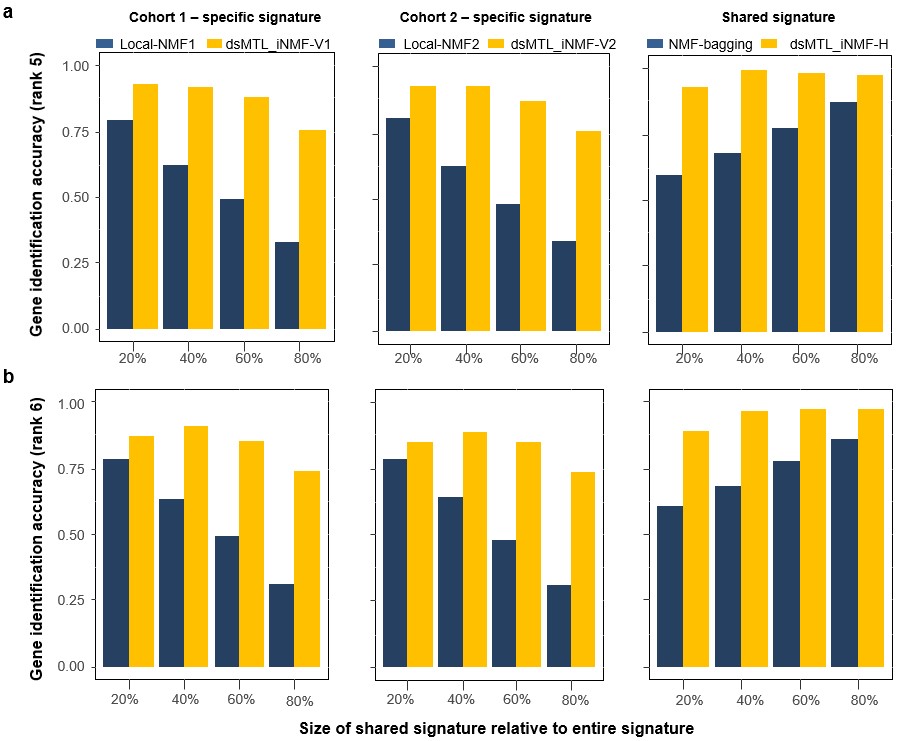

Supplement: btac616_Supplementary_Data [file btac616_supplementary_data.zip › Supplementary Figure 5.jpg]
